# Supplementary material for: Vespakinin-M, a natural peptide from Vespa magnifica, promotes functional recovery in stroke mice
Source: Commun Biol. 2022 Jan 20;5:74. doi: 10.1038/s42003-022-03024-5 (PMC8776894; doi:10.1038/s42003-022-03024-5)
Supplement: Supplementary file 2 — Supplementary Information [file 42003_2022_3024_MOESM2_ESM.pdf]

Supplementary Information for

Vespakinin-M, a natural peptide from *Vespa magnifica*, promotes functional recovery in stroke mice

Hairong Zhao et al.

Supplementary Methods

Platelet aggregation function test. Venous blood from (normal healthy rabbits) was collected in 3 mL sodium citrate anticoagulation vacuum plastic tubes. The collected venous blood was centrifuged for 10 min at 100 × g at 4°C to obtain the upper plasma, and the platelet rich plasma (PRP) was prepared, centrifuged again for 10 min at 2000 × g at 4°C to draw the upper plasma, the platelet poor plasma (PPP) was prepared and the number of platelets were measured in five categories using a hemocytometer. PPP was adjusted with PRP to a platelet concentration of 3×10^8 cells/ml was counted. The adjusted PRP was placed into a four-channel platelet aggregation instrument (Agg RAM, Helena Laboratories, USA) to determine the 5 min maximum platelet aggregation rate with adenosine diphosphate (ADP), arachidonic acid (AA), thrombin, collagen (COL) , and platelet-activating factor (PAF) or saline as inducers (at a final concentration of 6.07 μmol/L, 0.23 mg/L, 0.91 U/mL, 3.03 μg/mL and 0.009 mg/mL, respectively).

Acute toxicity test. Systemic acute toxicity test by peritoneal injection was performed according to the guideline provided by Center for Drug Evaluation and Research. In addition to the normal group, male mice were randomly divided into the treated groups received a single dose of 1.5 mg/kg, 6 mg/kg, 24 mg/kg, 96 mg/kg and 384 body weight of VK. Clinical signs of toxicity such as mortality, skin, eyes, fur, respiratory pattern , changes in general behavior and activity were individually observed. After observed 14 day, the mice were anesthetized with with 1.5% isoflurane in a mixture of 30% O_2 and 69% N_2O. Blood was collected from muse eyeballs and mixed with 3.2% sodium citrate at a v/v ratio of 1:9. Then, the blood was centrifuged at 3,000 rpm for 10 min. The obtained plasma was analyzed using an automatic coagulation apparatus (CA1500).

Histological analysis. Organs such as heart, liver, kidney, and brain were excised and fixed in 10% formaldehyde for histological analysis. Their organs were collected and fixed with 4% PFA for 12-18 h, followed by dehydrating using an automatic dehydrator (Leica ASP-300S, Germany) and embedding in paraffin. Then, each sample was coronally cut (4 μm) using a rotatory microtome (Leica RM2245, Germany). The sections were subsequently stained with H&E. Each section was observed at ×2 or ×40 magnification.

Primary culture of neuron and microglia. Primary cortical neurons (1.0×10^5 cells per well in poly-L-lysine-coated 24-well plate) were obtained from fetal C57BL/6 mice of embryonic day 16–17.5, as previously reported[1]. After careful removal of meninges, cortical tissues were digested in dulbecco's modified eagle medium (DMEM, Gibco) containing 0.05% trypsin (Gibco) at 37 °C for 20 min. DMEM containing 10% FBS (Gibco) was then added to terminate the digestion. The mixture was homogenized by pipetting 120 times and was subsequently filtered with a 100-μm cell strainer (Biologix) and centrifuged at 1000 rpm for 5 min. Then, the cells were collected and resuspended by DMEM supplemented with 10% FBS (Gibco) and penicillin-streptomycin (Gibco). The suspension was seeded into 24-well plates (10^5 cells/ml), and 4 h later, the medium was changed with Neurobasal medium (Gibco) containing 1% L-Glutamate and 2% B27 supplement. At the third day, 1/2 of the medium was changed and the neurons were used at the sixth or seventh day.

As reported[2], mixed glial cultures were isolated from WT mice at postnatal day 1–3 and were plated onto poly-l-lysine-coated flasks and grown in DMEM supplemented with 10% fetal bovine serum (FBS) (Gibco). After 3 days, the medium was changed to that

which contained 25 ng/ml granulocyte-macrophage colony stimulating factor) (GM-CSF, biolegend, 576306) and 10% FBS. Primary microglial cells were harvested by shaking (200 rpm) after 10–12 days in culture and once every 3 days thereafter (up to four harvests).

For co-culture experiments, primary microglia in 200 μL (10^6 cells) HUA with serum-free DMEM medium configurations were loaded into the upper chamber of the transwell insert. Primary neuron were grown in bottom plates of the transwell system with 600 μL DMEM medium containing 15% FBS. After 24 h, medium in the transwell inserts was carefully removed. Primary neuron were assayed for neuroinflammation by Elisa.

Cell cultures, treatment, and OGD. BV2 microglia cells and HT22 hippocampal neuron cells were routinely cultured with complete medium (DMEM containing 10% FBS, 100 U/mL penicillin G, and 100 mg/mL streptomycin) in a conventional incubator (37°C, 5% CO_2, unlimited O_2 content). Experiments were divided into the four treatment groups: control group, OGD group, and VK groups (0.0735 μM, 0.735 μM). To establish the OGD/R model, BV2 cells or HT22 cells were incubated with DMEM free of glucose and FBS and placed in an anaerobic incubator defined as 1% O_2, 5% CO_2, and 94% N_2, at 37°C. After 6 h of OGD, cells were changed to complete medium and reoxygenated in a conventional incubator for 18 h.

CCK-8 assay. Cell viability was measured by the CCK-8 assay. In detail, BV2 and HT22 cells were seeded on 96-well plates and treated with different concentrations of VK (0.0735 μM, 0.735 μM, 7.35 μM, 73.5 μM, 735 μM). Cells treated with DMSO (0.1%, v/v) acted as the vehicle control. After 24 h of incubation, the OGD/R model was established and 10 μL of the CCK-8 regent were added to each well. The plate was

incubated at 37°C for 4 h. Absorbance was surveyed at 450 nm by a Multiscan Spectrum (Spark, Tecan, Switzerland).

Measurement of intracellular ROS levels. HT22 cells sub-cultured in 12-well plates (5×10^5 cells/well) were allowed to attach for 24 h, pretreated with VK for 24 h, then performed OGD/R and stained with 10 nM DCFH-DA for 30 min at 37 °C as described [3]. Stained cells were analyzed by flow cytometry at emission 480 nm and excitation 530 nm. Rosup (S0033S-2), a positive control, was used to prove experimental feasibility.

qRT-PCR with reverse transcription analysis. After OGD/R, BV2 cells were treated with VK (0.08 μM, 0.8 μM) for 24 h. Total cellular RNA was purified by using an RNeasy Mini Kit (Qiagen) and cDNA was synthesized by using the iScript DNA Synthesis Kit (Bio-Rad). The resulting complementary DNA was examined by qRT-PCR with reverse transcription with SYBR Green PCR Master Mix (Applied Biosystems). The GAPDH housekeeping gene was used to normalize samples. Quantitative analysis was performed with the $^{\Delta\Delta}$CT method. The primers (5′–3′) used were designed on Primer-Blast and are shown in Table 4.

Annexin V-FITC/propidium iodide. After OGD/R, HT22 were treated with VK (0.08 μM, 0.8 μM) for 24 h. The FITC Annexin V Apoptosis Detection Kit (#556419) was purchased from BD Biosciences. HT22 (1×10^5/well) cells were stained with this kit following the manufacturer instructions (https://www.bdbiosciences.com/en-us/products/reagents/flow-cytometry-reagents/research-reagents/single-color-antibodies-ruo/fitc-annexin-v.556419).

Immunohistochemistry. IHC staining was conducted to identify the expression of microglia and neurons. Briefly, antigen retrieval was carried out by heating the tissue in 10 mM sodium citrate buffer at pH 6.0 in a microwave. These sections were

incubated for 1 hour in the blocking solution (0.1% Triton-X, 10% normal goat serum in 1X PBS) at room temperature. The primary anti-IBA-1 (1:400, 019-19741, WAKO, Japan) and anti-NeuN (1:400, ab177487, Abcam) were respectively added to each section and placed overnight at 4°C. The samples were then incubated through the use of biotin-labeled secondary antibodies at room temperature for 30 min. The HRP-labeled SP working medium was added and incubated at room temperature for 30 min. The positive signals of protein expression of IBA-1 and NeuN were localized to cell cytoplasm, which manifests as pale yellow, brown, or sepia positive granules.

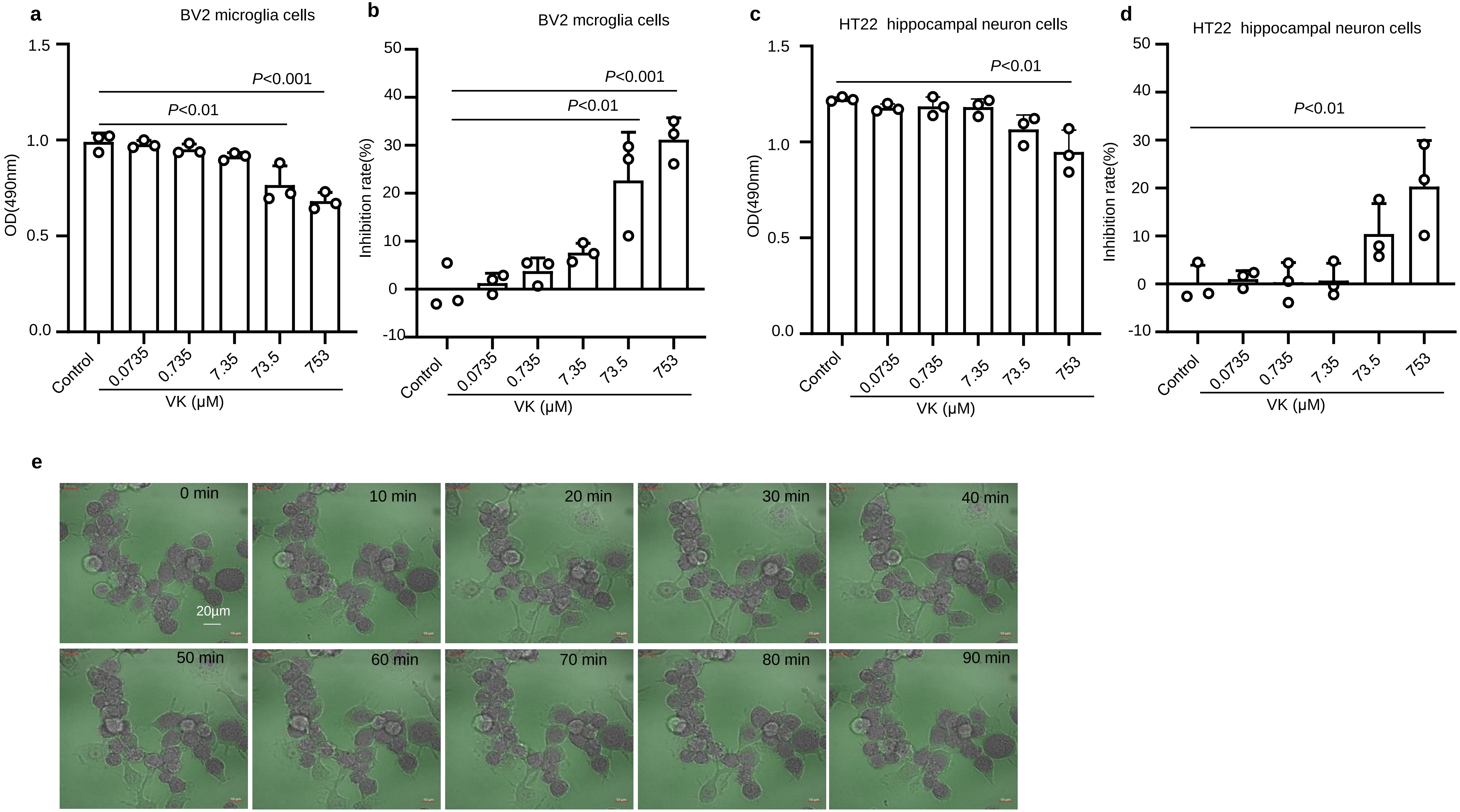
a
BV2 microglia cells
OD(490nm)
P<0.001
P<0.01
Control
0.0735
0.735
7.35
73.5
753
VK (μM)
b
BV2 mcroglia cells
Inhibition rate(%)
P<0.001
P<0.01
VK (μM)
c
HT22 hippocampal neuron cells
OD(490nm)
P<0.01
VK (μM)
d
HT22 hippocampal neuron cells
Inhibition rate(%)
P<0.01
VK (μM)
e
0 min
10 min
20 min
30 min
40 min
50 min
60 min
70 min
80 min
90 min
20μm

Supplementary Fig. 1 The cytotoxicity or transmembrane of VK on microglia BV2 cells or neuron HT22 cells. a–d, The effects of VK on microglia BV2 cells or neuron HT22 cells. **e**.Live cell imaging by FITC-treated was collected after OGD/R .

a

Concentration (μM)	Platelet aggregation rate (%)				
	ADP	PAF	AA	COL	Thrombin
0.294	52.97±4.75	62.37±5.10	62.80±14.34	64.63±4.95	73.67±4.22
0.147	44.37±6.93	59.80±4.25	61.77±12.51	64.30±2.18	73.33±6.13
0.0735	46.83±6.75	59.90±7.86	62.27±13.91	66.27±6.04	74.73±6.03
0.0368	50.03±7.05	62.37±4.95	61.33±14.35	64.10±5.79	73.10±11.09
0.0184	49.20±7.03	59.97±3.89	66.13±12.82	67.33±6.36	48.93±34.34
0.0091	46.77±5.26	59.63±11.35	65.00±11.05	64.20±5.65	50.77±32.91

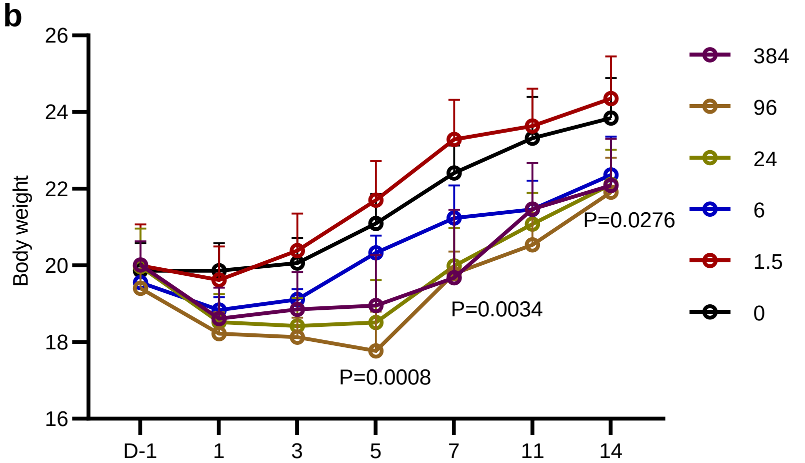

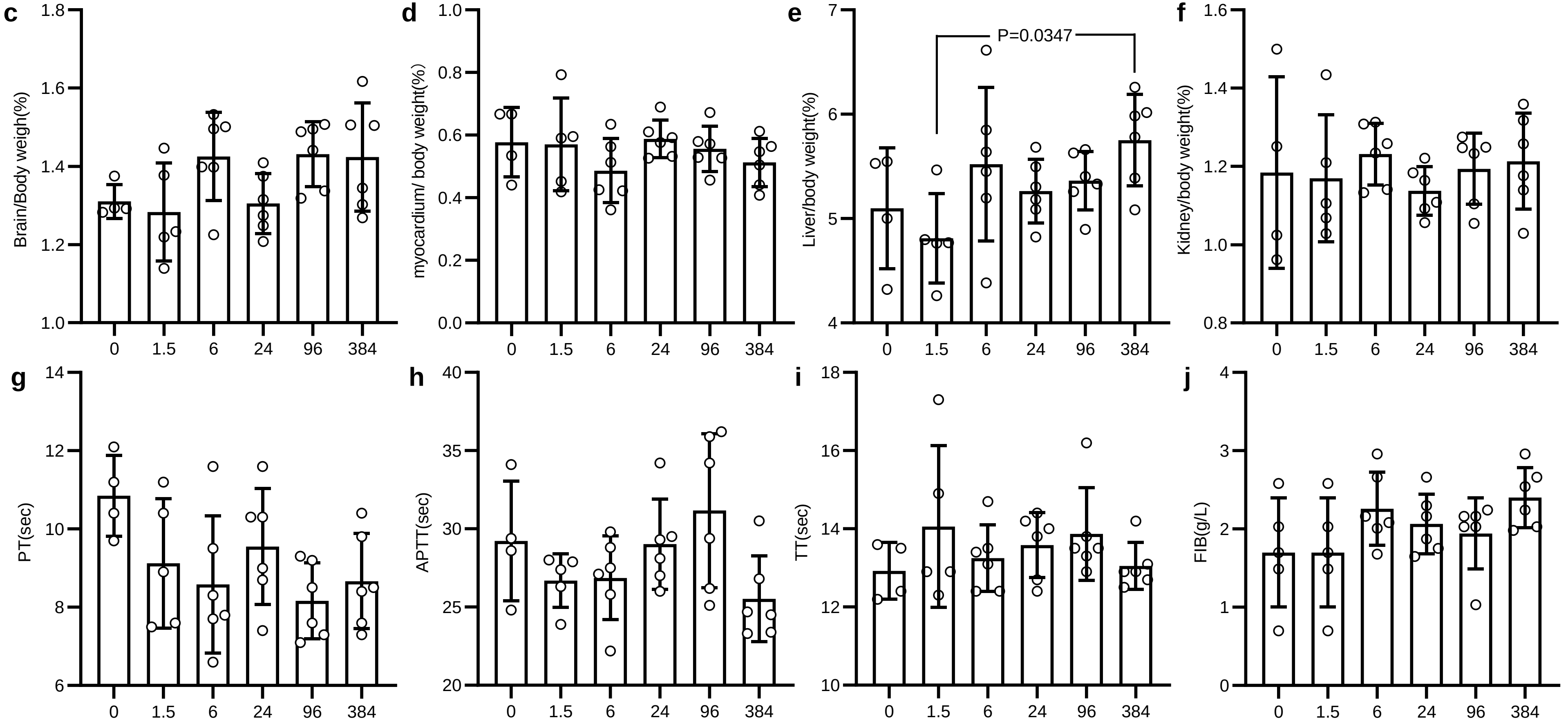

Supplementary Fig. 2 The effects of VK on platelet aggregation function, body weight, coagulation and organ index in mice. a Platelet-activating factor(PAF),arachidonic acid (AA), adenosine diphosphate (ADP), thrombin, and collagen (COL) or saline as inducing agents was employed to evaluate effect of VK on platelet aggregation function in vitro. **b** Body weight assessment of VK-treated (1.5,6,24,96 and 384 mg/kg) mice in acute toxicity study. Weight index of brain **c**, myocardium **d**, liver **e**, kidney **f** was assessed. g-j Coagulation function such as prothrombin time (PT), activated partial thromboplastin time (APTT), thrombin time (TT) and plasma fibrinogen (FIB) of mice treated with different dose level of VK in acute toxicity after 14th day. n=4-6.

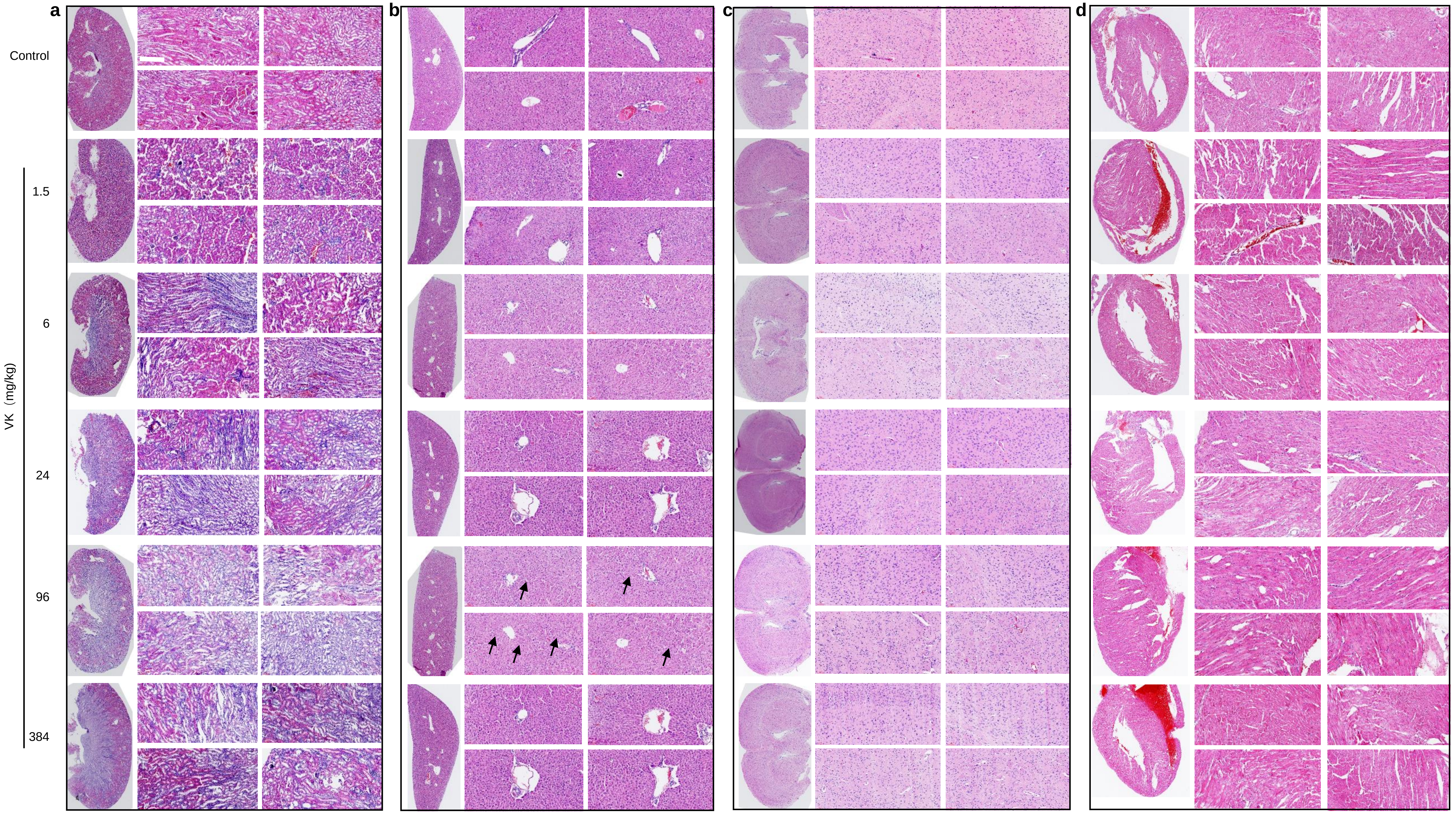
a
b
c
d
Control
1.5
6
24
96
384
VK (mg/kg)

Supplementary Fig. 3 Histopathology with H&E staining of VK-treated groups in mice after ^{14}th days. **a** Kidney. **b** Liver. **c** Brain (Interaural 4.66 mm; Bregma 0.86 mm). **d** Myocardium. The magnified areas evaluated under ×20 magnification. Scale bars=20 μm, n=4-6.

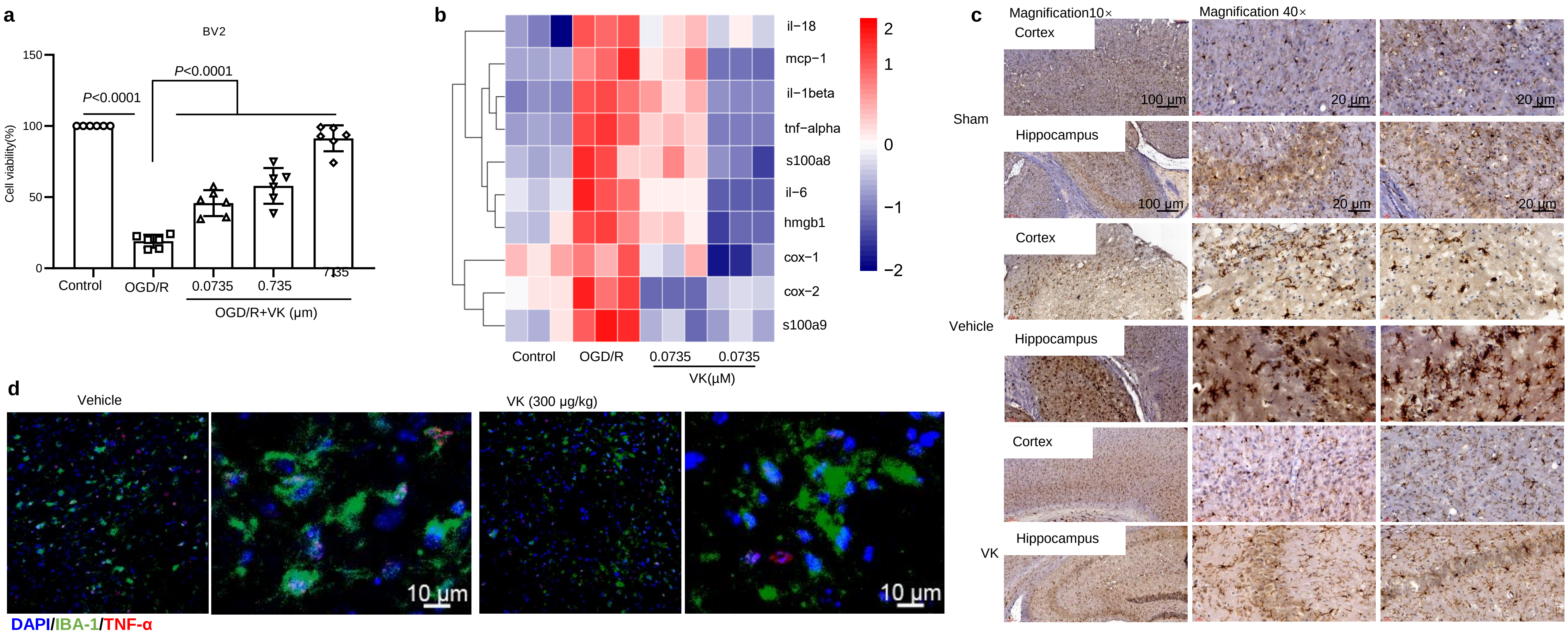
a
BV2
Cell viability(%)
150
100
50
0
P<0.0001
P<0.0001
Control
OGD/R
0.0735
0.735
7.35
OGD/R+VK (μm)
b
il−18
mcp−1
il−1beta
tnf−alpha
s100a8
il−6
hmgb1
cox−1
cox−2
s100a9
2
1
0
−1
−2
Control
OGD/R
0.0735
0.0735
VK(μM)
c
Magnification10×
Magnification 40×
Sham
Vehicle
VK
Cortex
Hippocampus
100 μm
20 μm
d
Vehicle
VK (300 μg/kg)
10 μm
DAPI/IBA-1/TNF-α

Supplementary Fig. 4 VK observably reduced the expression of pro-inflammatory mediators and inhibited microglia activation. **a** Cell viability in BV2 cells after oxygen glucose deprivation/reoxygenation (OGD/R) was investigated by the CCK8 assay. **b** Proinflammatory mediators were detected by RT-PCR, the heat map represents fold change of relative mRNA expression. Data are mean ± SD. **c** IHC of brain sections and bar graph showing relative Iba1 positive area compared to the sham group. Brown represents Iba1, a marker of microglia. Scale bar =100 μm or 20 μm. **d** TNF-α co-localization with Iba-1 was performed by immunofluorescence, Blue: DPAI, Green: Iba-1,Red: TNF-α. Scale bar =10 μm. Data are mean ± SD.

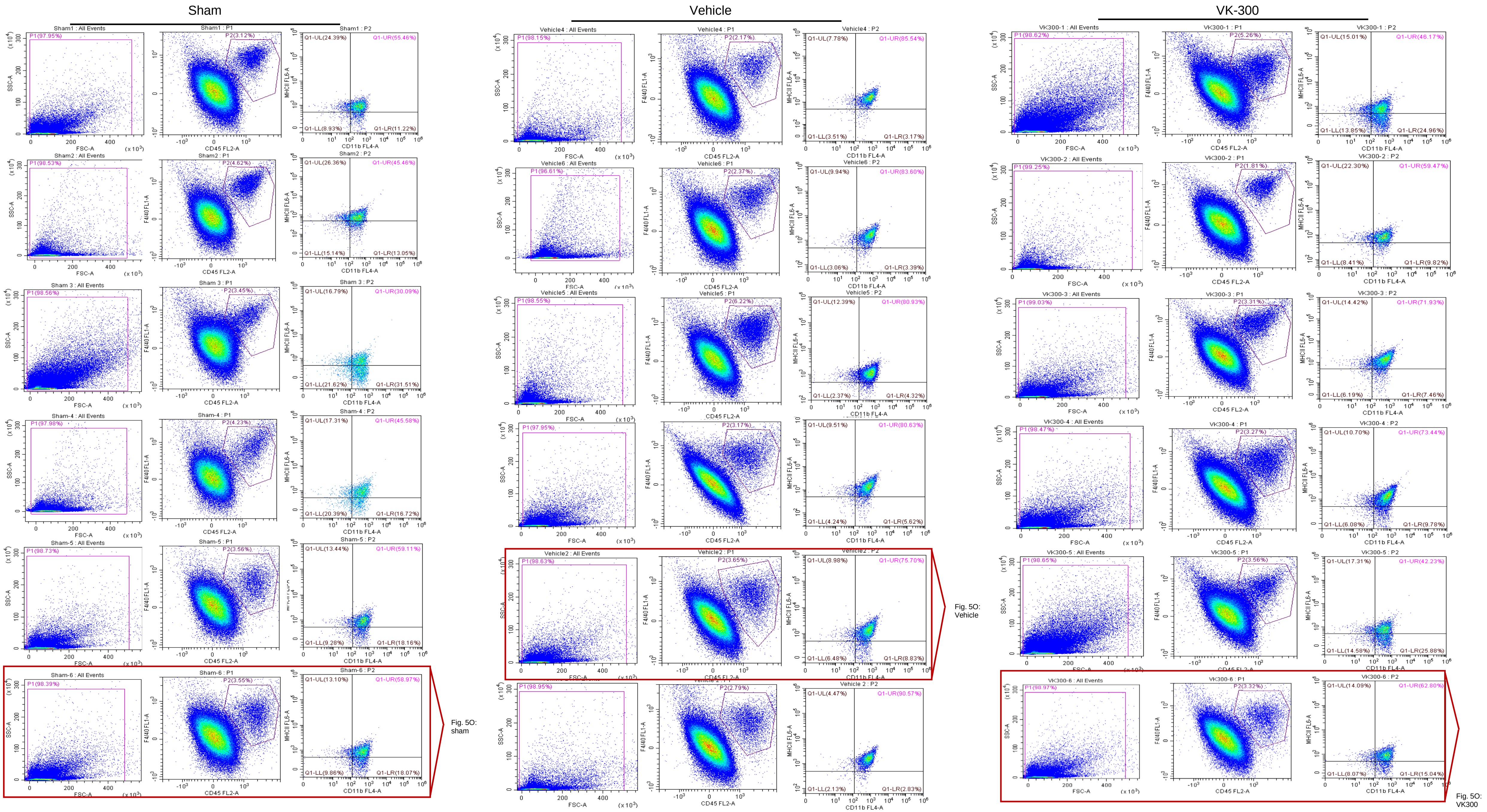
Sham
Vehicle
VK-300
Fig. 5O: sham
Fig. 5O: Vehicle
Fig. 5O: VK300

Supplementary Fig 5: The FACS gating strategy and original data referring to Fig. 5O.

FACS gating strategy: P1=all live cells,P2=CD45+F4/80+=Microglia from P1,

Q1 UR=CD45+F4/80+CD11b+MHCⅡ+ from P2.

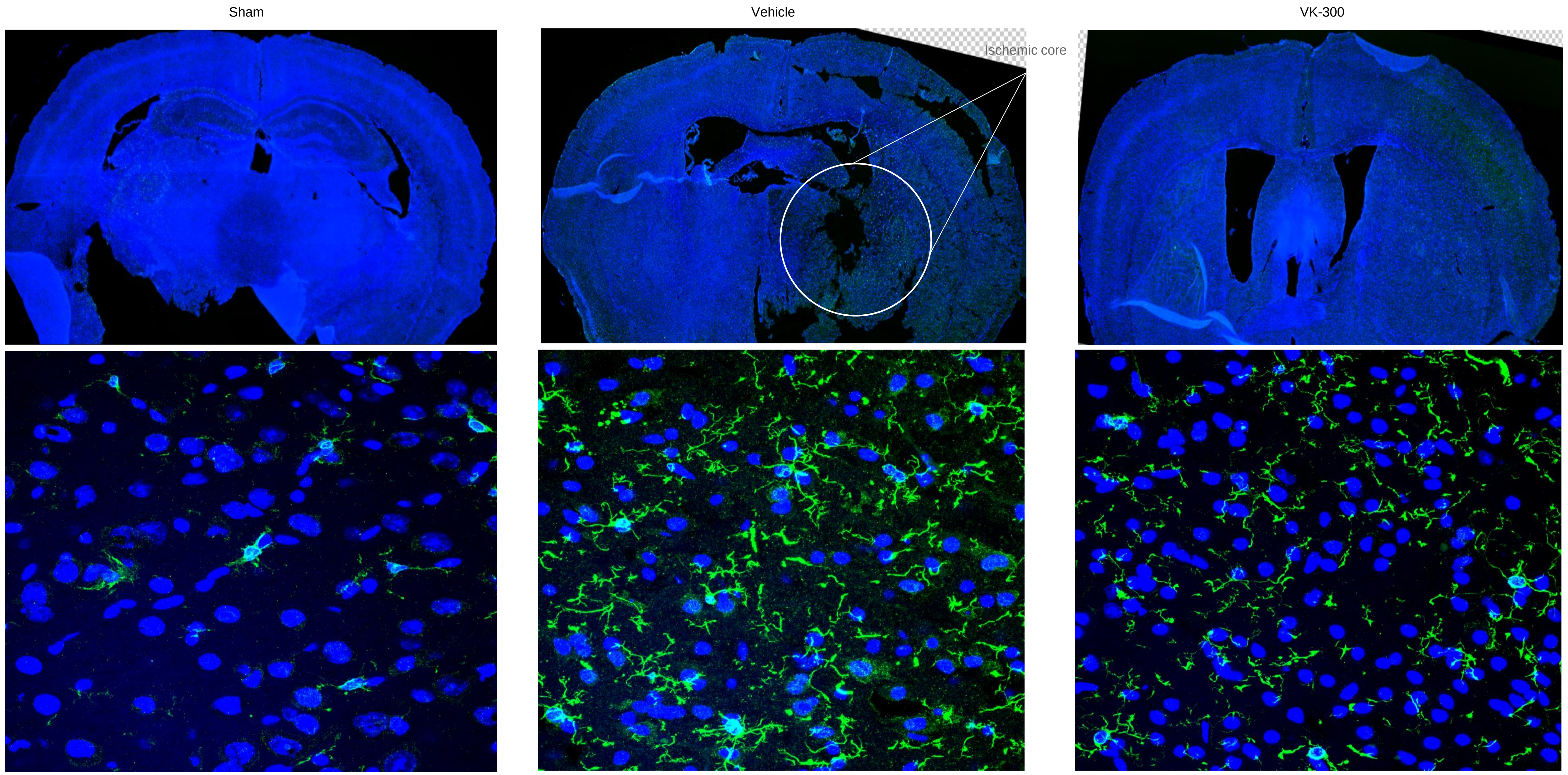

Supplementary Fig 6: The panoramagram of immunofluorescence referring to Fig. 5l.

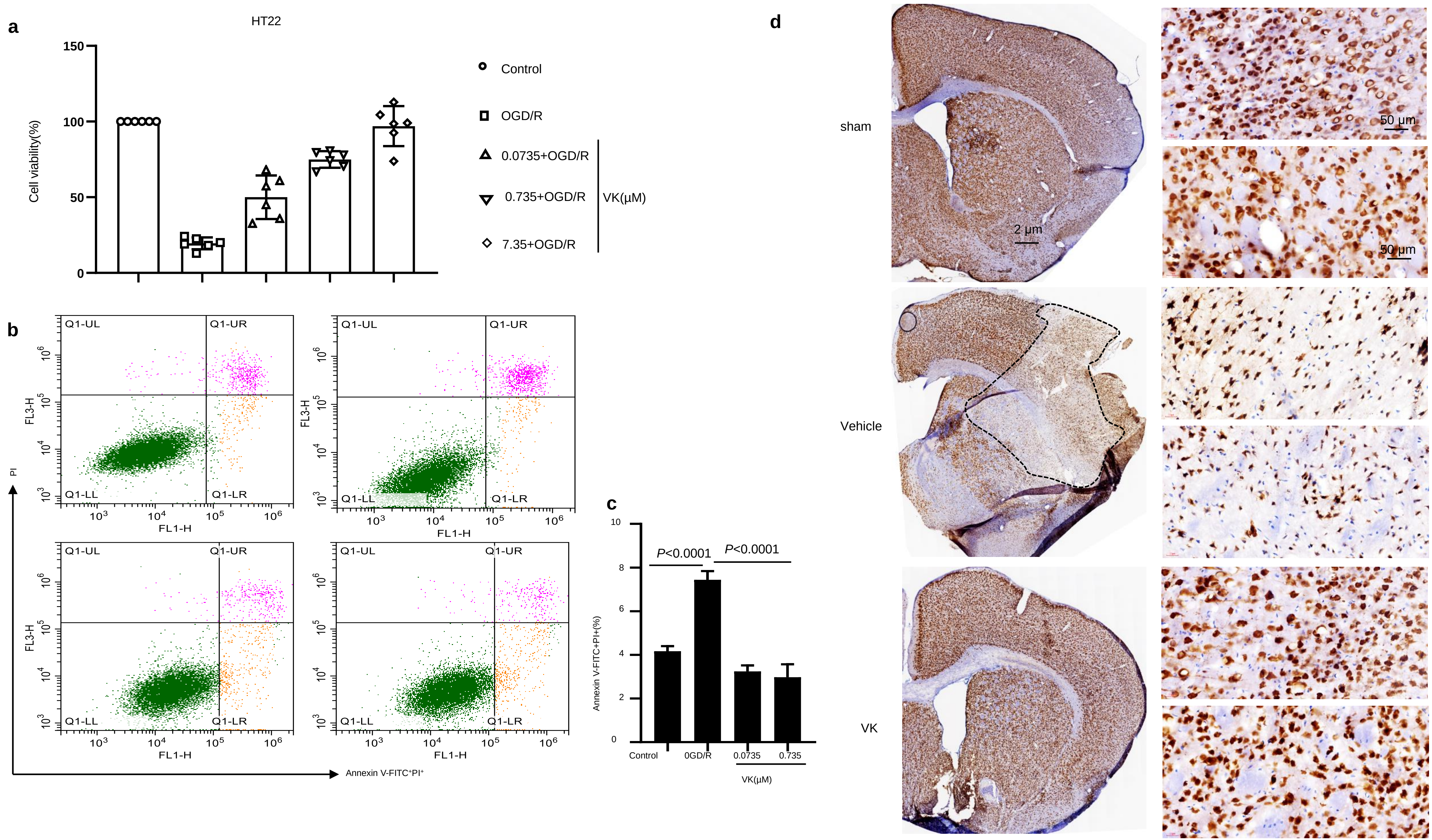
a
HT22
Cell viability(%)
150
100
50
0
Control
OGD/R
0.0735+OGD/R
0.735+OGD/R
7.35+OGD/R
VK(μM)
b
Q1-UL
Q1-UR
Q1-LL
Q1-LR
FL3-H
FL1-H
PI
Annexin V-FITC⁺PI⁺
c
Annexin V-FITC+PI+(%)
P<0.0001
P<0.0001
Control
0GD/R
0.0735
0.735
VK(μM)
d
sham
Vehicle
VK
2 μm
50 μm
50 μm

Supplementary Fig. 7 VK inhibited neuronal apoptosis. a Cell viability in HT22 cells after oxygen glucose deprivation/reoxygenation (OGD/R) were investigated by CCK8 assays. **b-c** Neuronal apoptosis was measured using a FITC Annexin V Apoptosis Detection Kit by flow cytometry. **d** IHC of brain sections and bar graph showing relative NeuN positive area compared to the sham group. Brown represents NeuN, a marker of neurons. Scale bar is 50 μm. Data are mean ± SD.

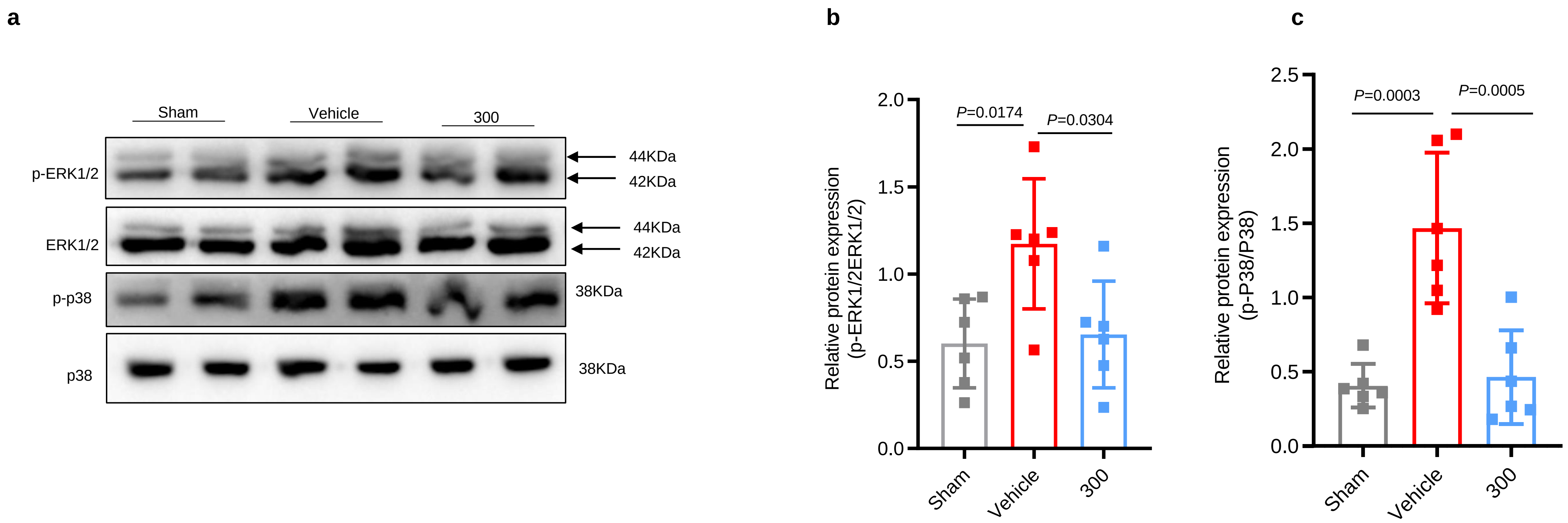
a
Sham
Vehicle
300
p-ERK1/2
ERK1/2
p-p38
p38
44KDa
42KDa
44KDa
42KDa
38KDa
38KDa
b
Relative protein expression (p-ERK1/2ERK1/2)
P=0.0174
P=0.0304
2.0
1.5
1.0
0.5
0.0
Sham
Vehicle
300
c
Relative protein expression (p-P38/P38)
P=0.0003
P=0.0005
2.5
2.0
1.5
1.0
0.5
0.0
Sham
Vehicle
300

Supplementary Fig. 8 VK treatment partially downregulated phosphorylation levels of p38 and ERK in stroke mice. a–c Western blot analysis revealed that there were significant changes in the phosphorylation levels of p38 and ERK in the VK groups compared to the vehicle group. Data are mean ± SD.

a

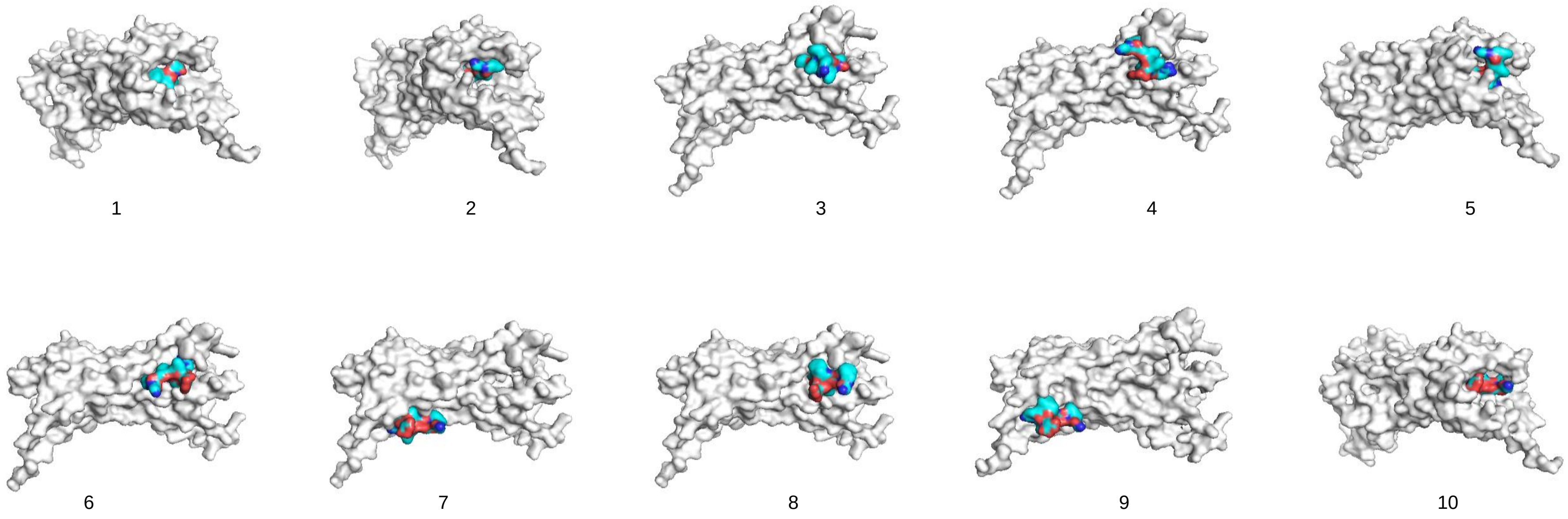

b

Summary of the Top 10 models (B1R)

	1	2	3	4	5	6	7	8	9	10
Docking Score	-229.28	-219.21	-208.22	-194.63	-192.73	-192.62	-188.41	-185.76	-182.79	-180.81
Ligand rmsd(Å)	15.44	9.36	30.31	30.35	18.73	27.78	19.84	31.41	22.91	14.18

c

Complex Template Information(B1R)

PDB ID	Chain ID	Align_length	Coverage	Seq_ID(%)
4XNV	A	292	0.874	22.9
4P9I	A	12	1	33.3

Supplementary Fig. 9 The peptide docking model of VK and B1R. **a** The peptide docking model of VK and B1R with the highest score (1-10) . The docking summary of the top 10 models is shown in **b**. The template information is listed below the NGL viewer c if available **c**.

a

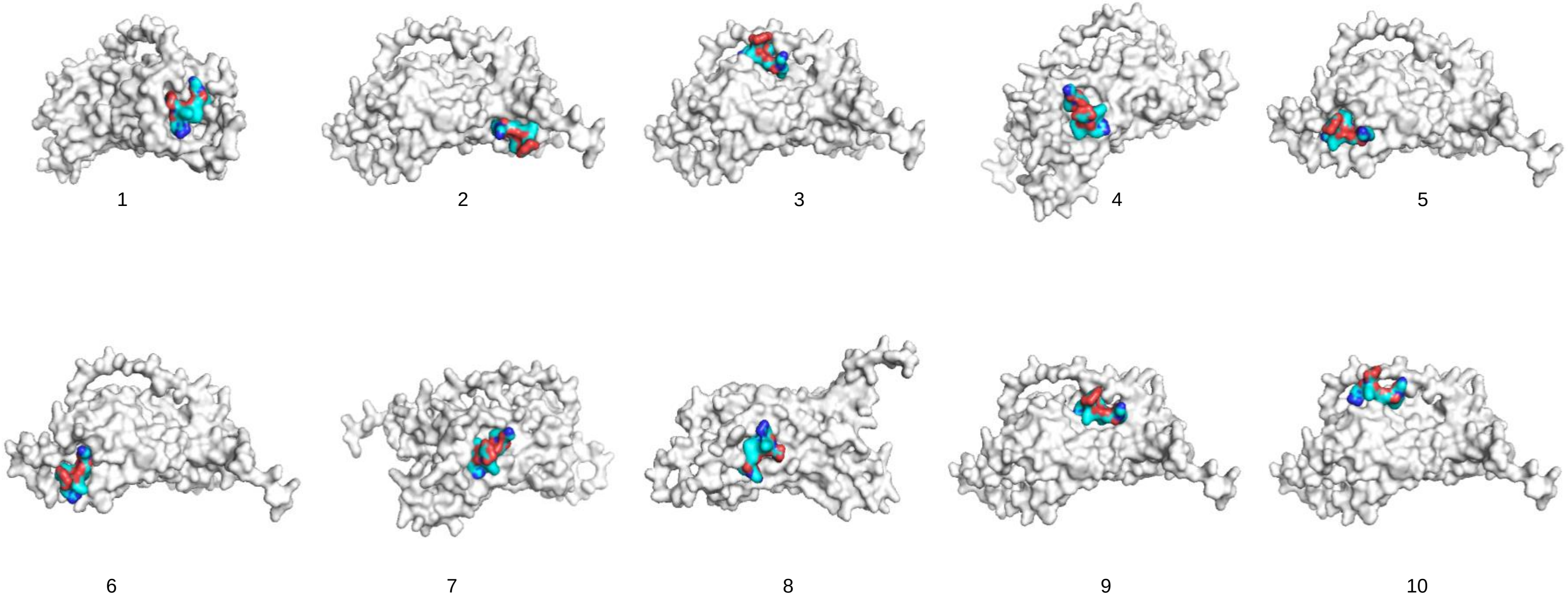

b

Summary of the Top 10 models (B2R)

	1	2	3	4	5	6	7	8	9	10
Docking Score	-212.17	-188.22	-187.84	-187.18	-183	-182.53	-182.45	-181.83	-181.02	-180.96
Ligand rmsd(Å)	22.85	32.32	18.05	23.23	29.23	30.72	21.64	22.7	19.35	20.67

Complex Template Information(B2R)

c

Molecule	PDB ID	Chain ID	Align_length	Coverage	Seq_ID(%)
Receptor	4XT1	A	317	0.809	28
Ligand	4P9I	A	12	1	33.3

Supplementary Fig. 10 The peptide docking model of VK and B2R. a The peptide docking model of VK and B2R with the highest score (1-10) . The docking summary of the top 10 models is shown in **b**. The template information is listed below the NGL viewer c if available **c**.

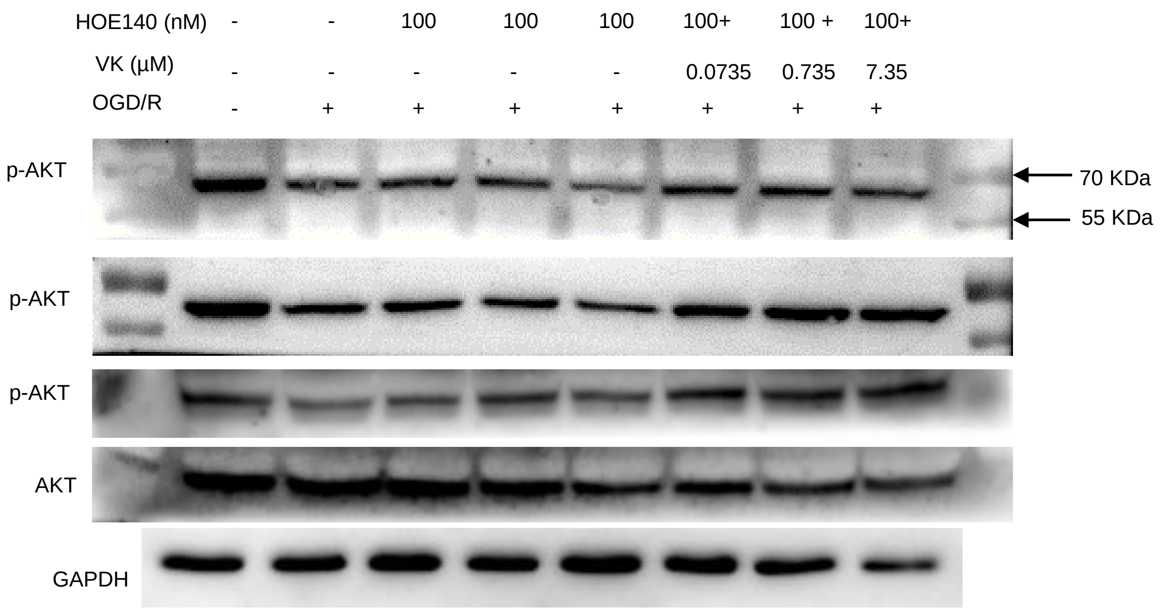

Supplementary Fig. 11 HOE140 could inhibit VK-mediated AKT activation.

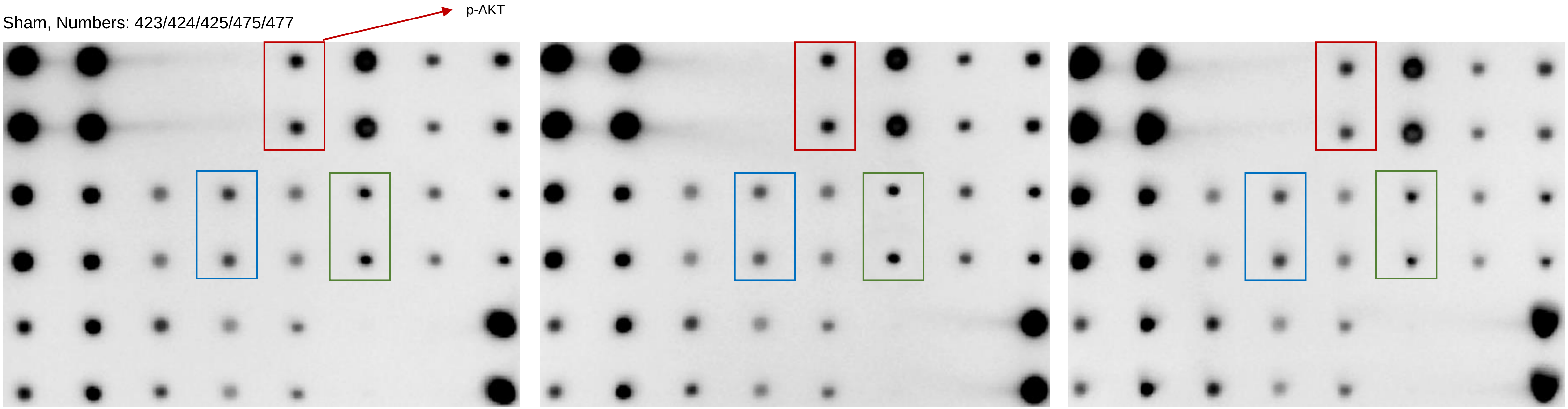

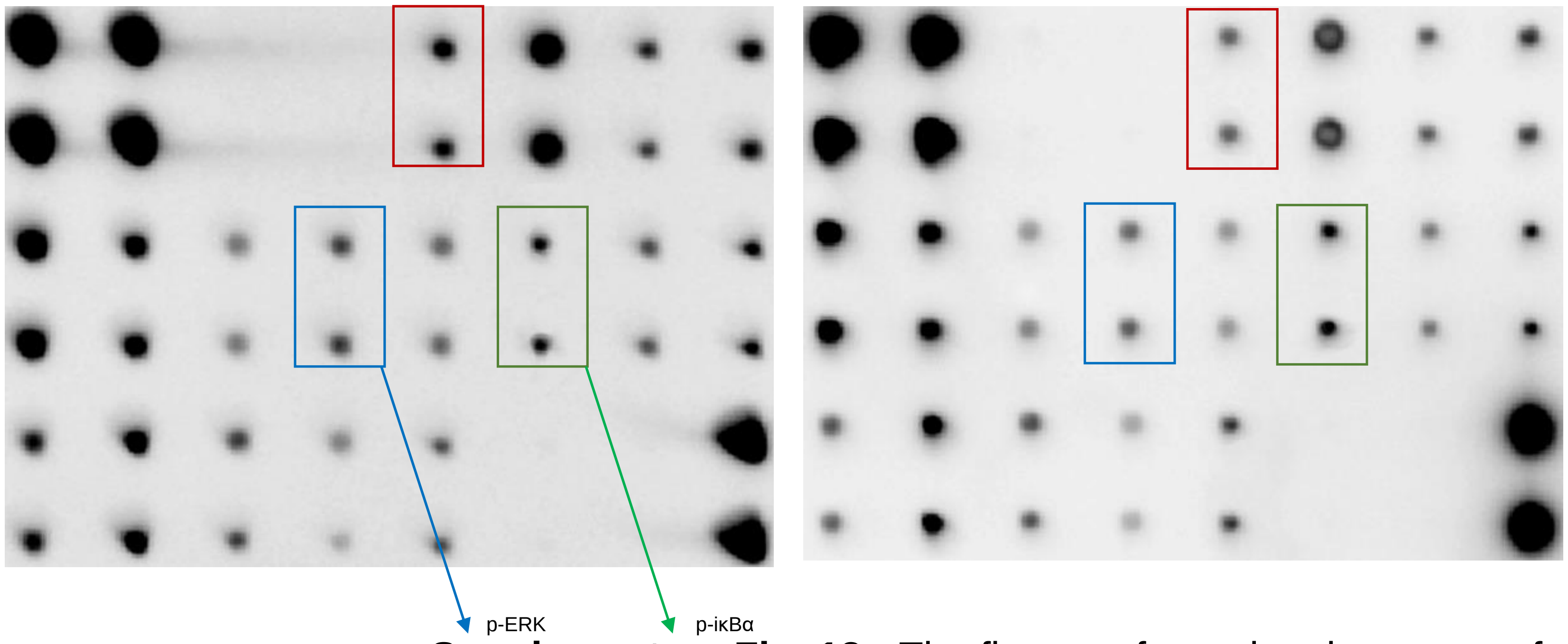

	a	b	c	d	e	f	g	h
1<br>2	POS	POS	NEG	NEG	AKT(S473)	ATM (S1981)	BAD (S112)	POS
3<br>4	Casp7 (D198)	CHK1 (S296)	eIF2a (S51)	Erk1/2 (T202)	Hsp27 (S82)	IkBa (S32)	JNK (T183)	Casp7 (D198)
5<br>6	P27 (T198)	P38(T180/Y182)	P53 (S15)	SMAD2 (S245)	TAK1 (S412)	Neg	Neg	POS

Supplementary Fig. 12: The figures of protein microarray referring to Fig. 7b:sham group.

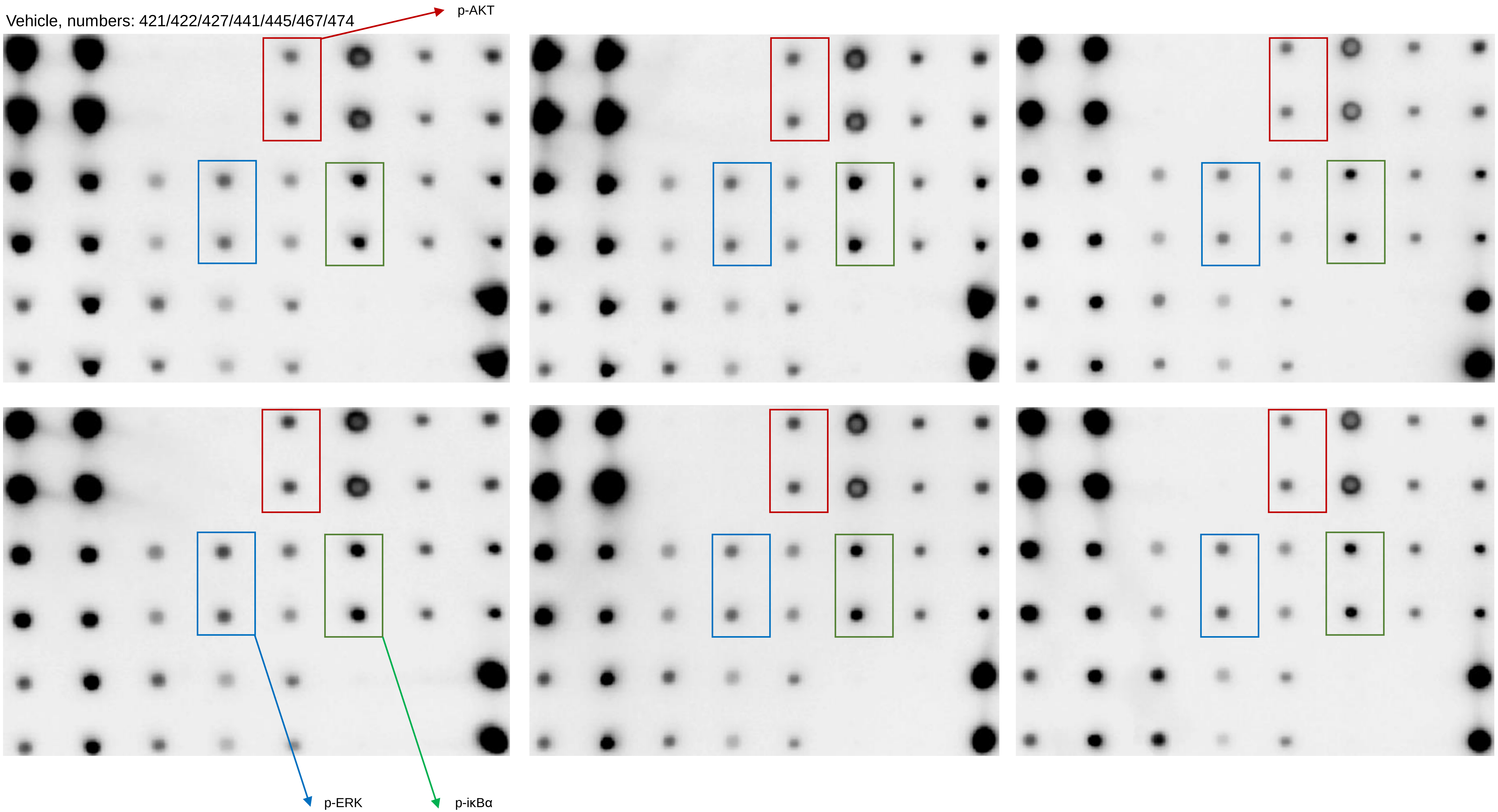

Supplementary Fig.13： The figures of protein microarray referring to Fig. 7b vehicle group。

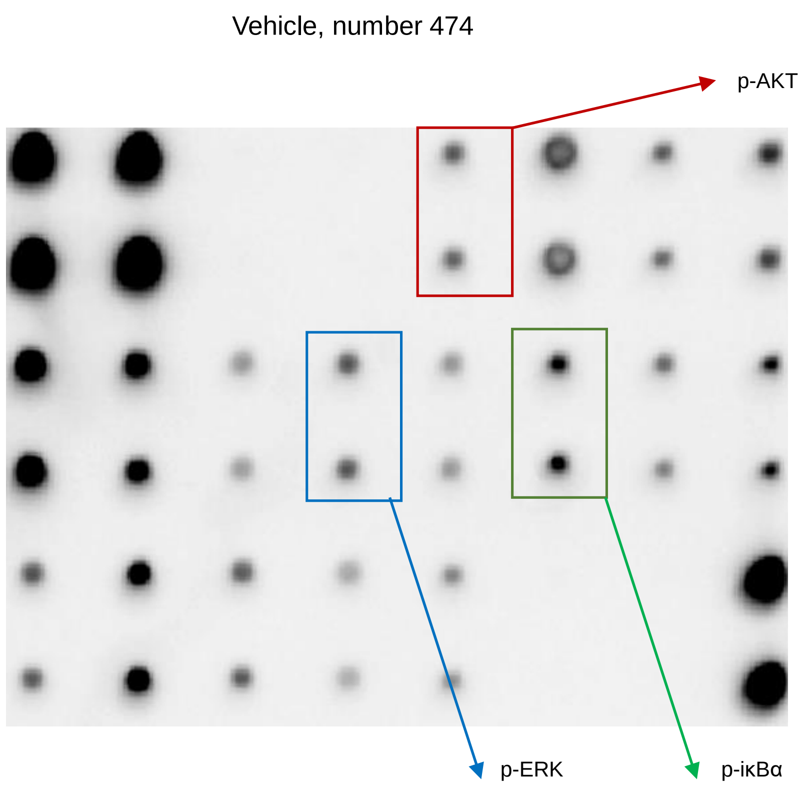

Supplementary Fig. 13: The figures of protein microarray referring to Fig. 7b vehicle group.

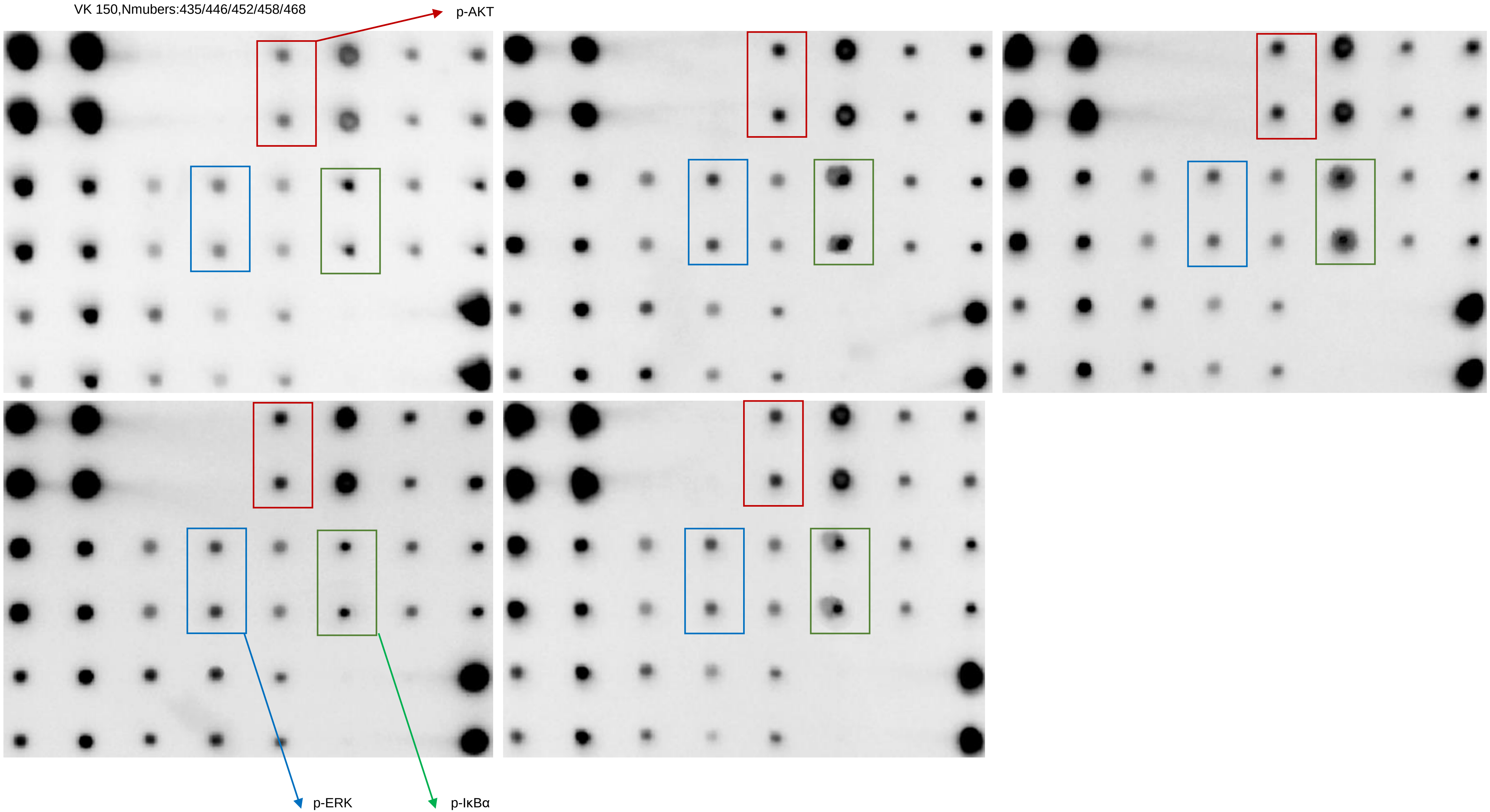

Supplementary Fig. 14: The figures of protein microarray referring to Fig. 7b VK150 group.

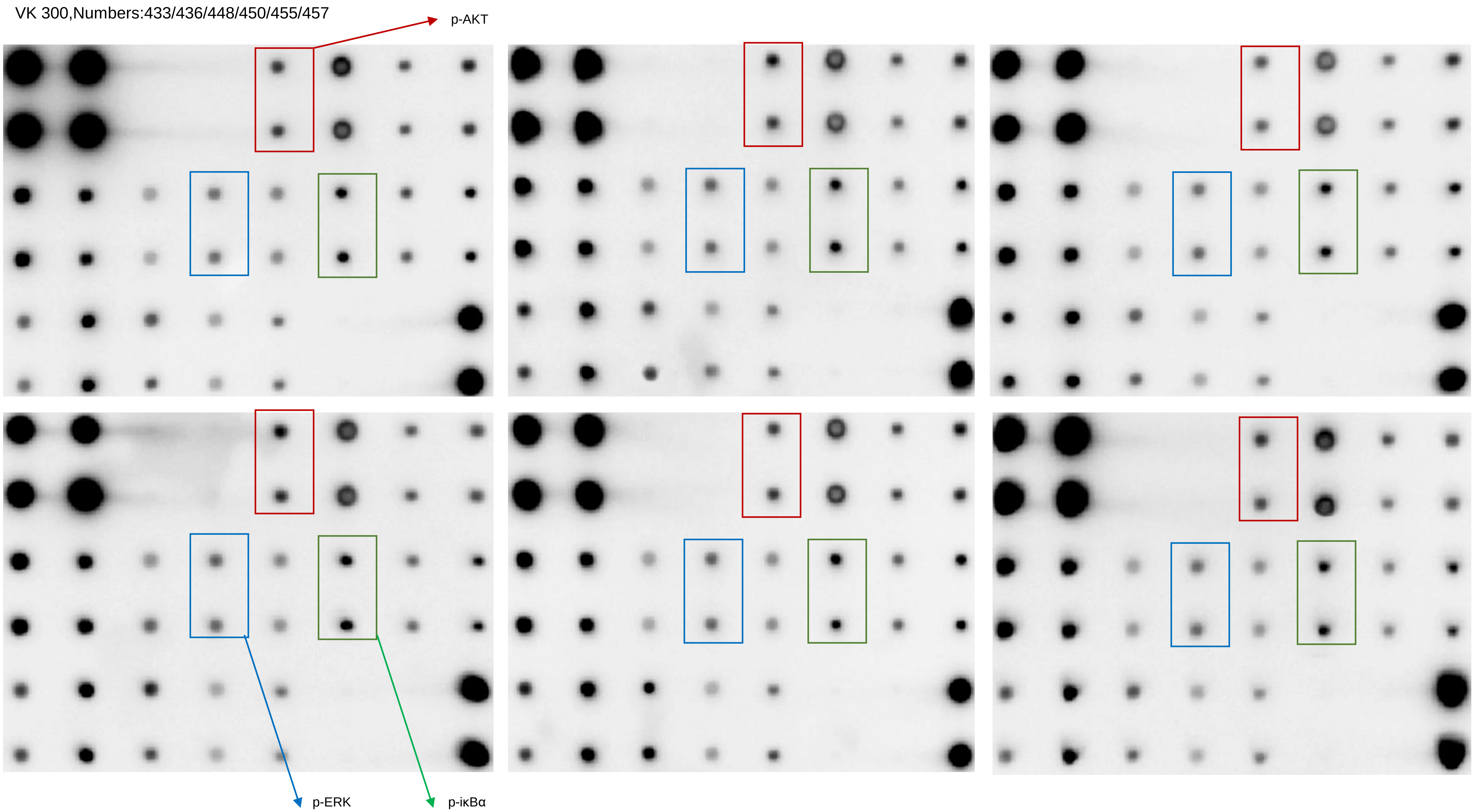

Supplementary Fig. 15: The figures of protein microarray referring to Fig. 7b VK300 group.

VK 300,Number:466

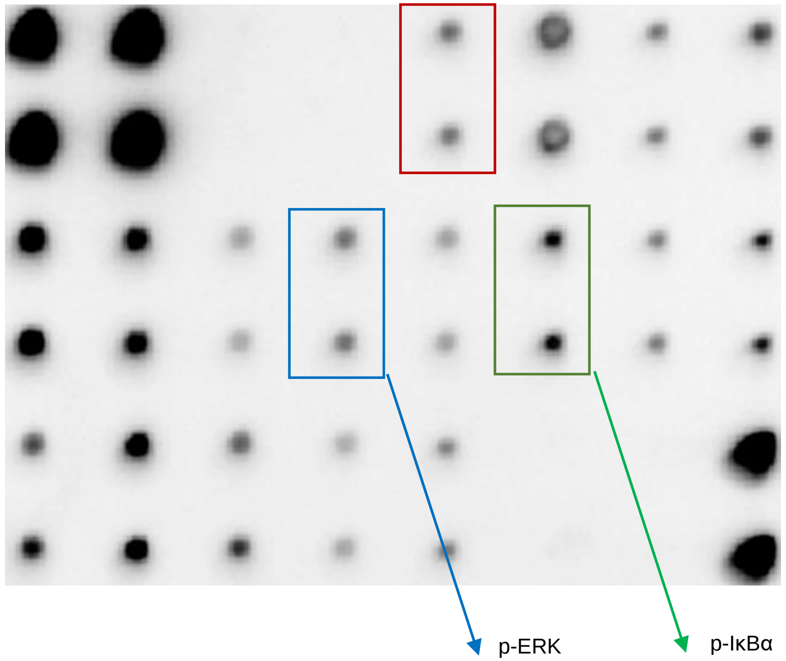

Supplementary Fig. 15: The figures of protein microarray referring to Fig. 7b VK300 group.

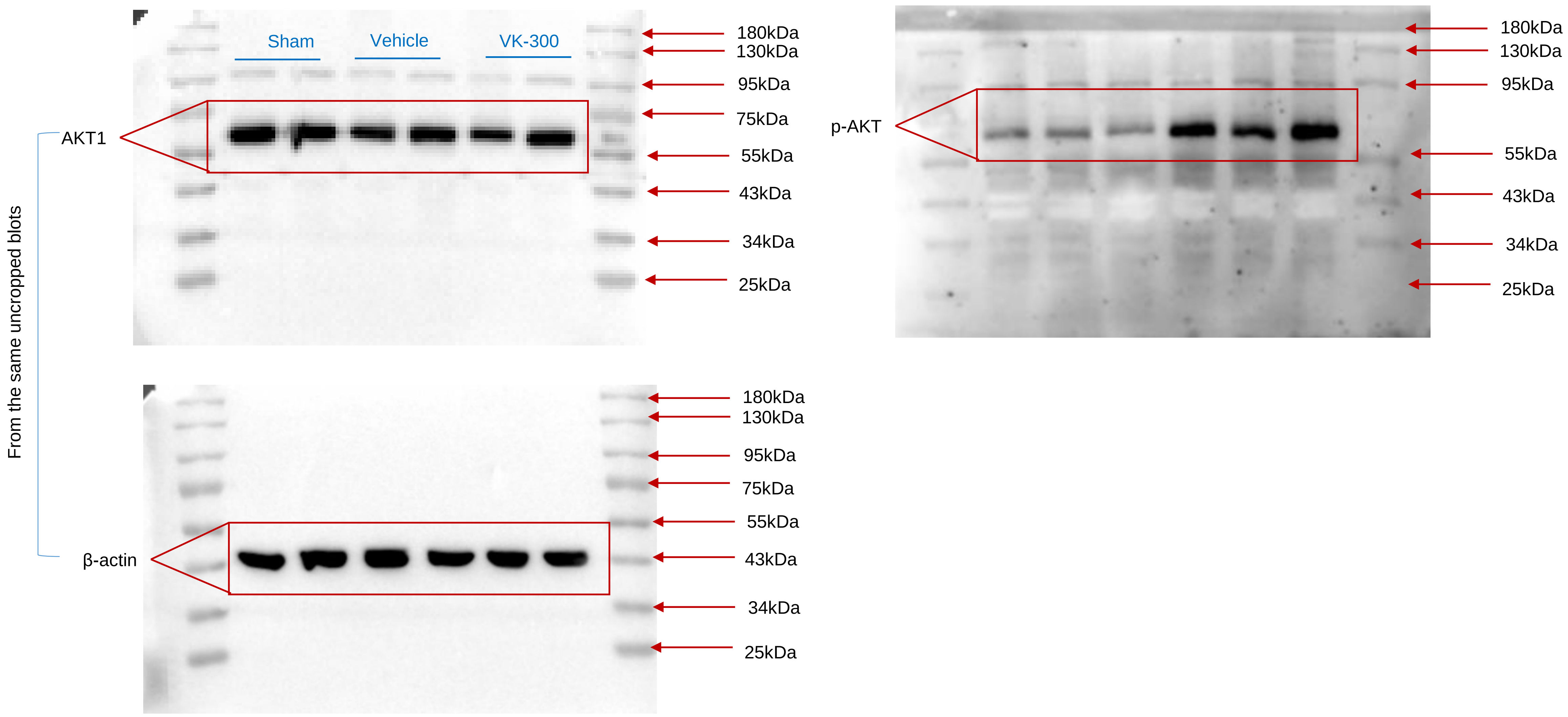

Supplementary Fig. 16: Uncropped blots referring to Fig. 7d.

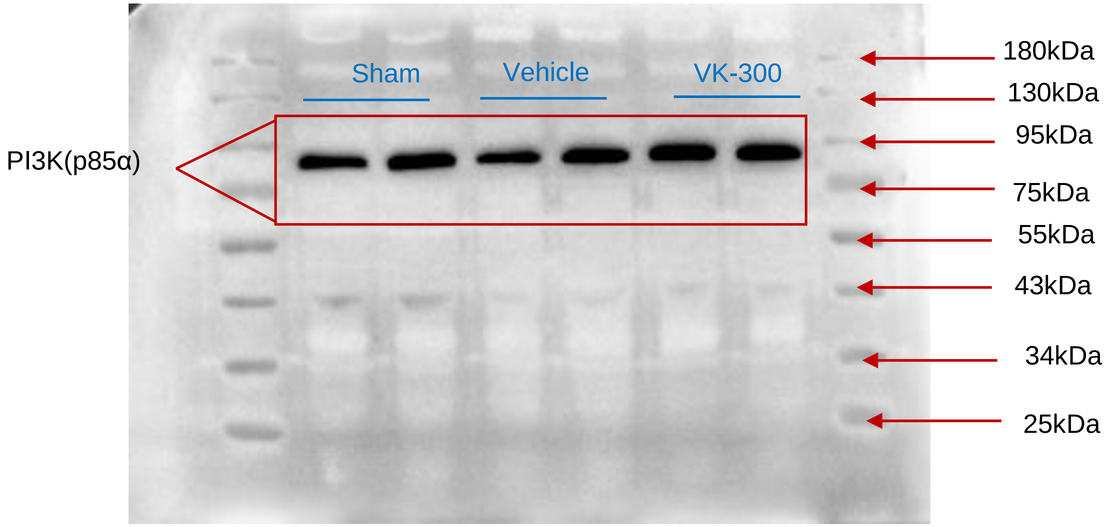

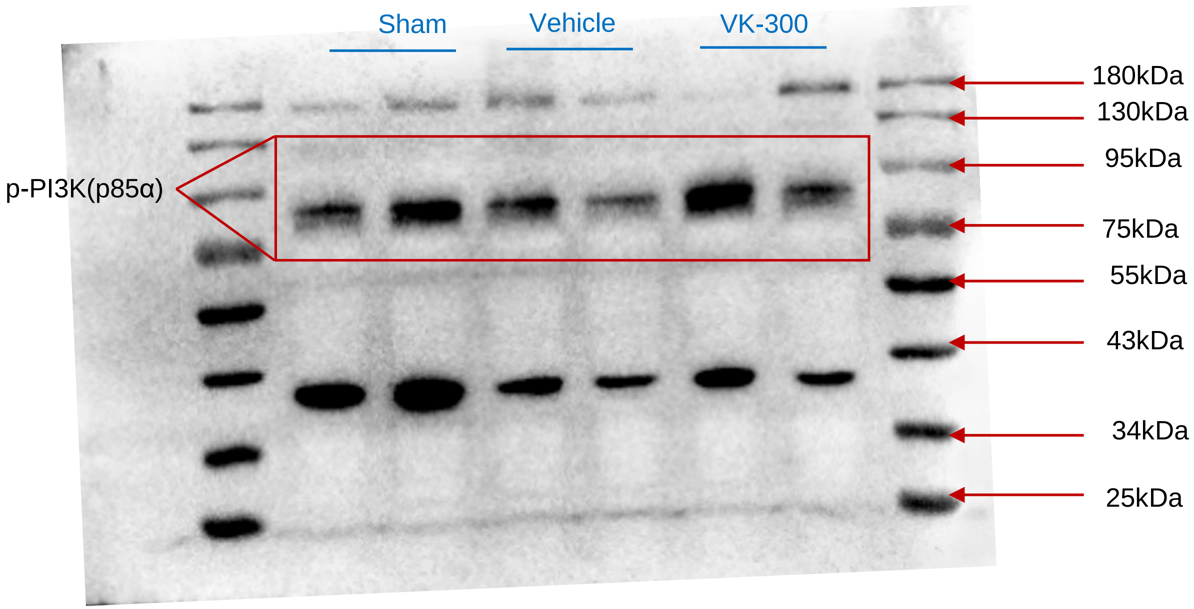

Supplementary Fig. 17: Uncropped blots referring to Fig. 7d.

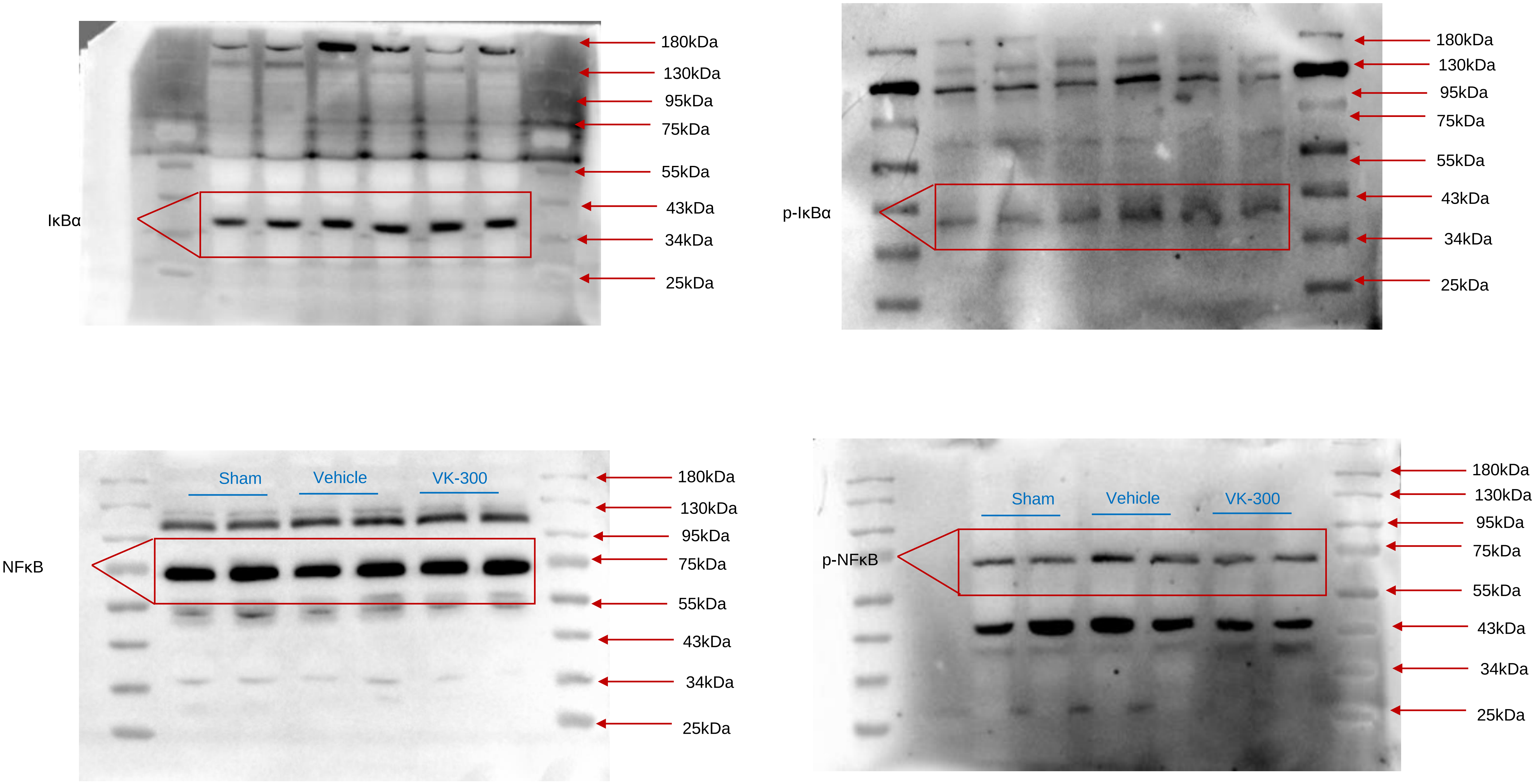

Supplementary Fig. 18: Uncropped blots referring to Fig. 7d.

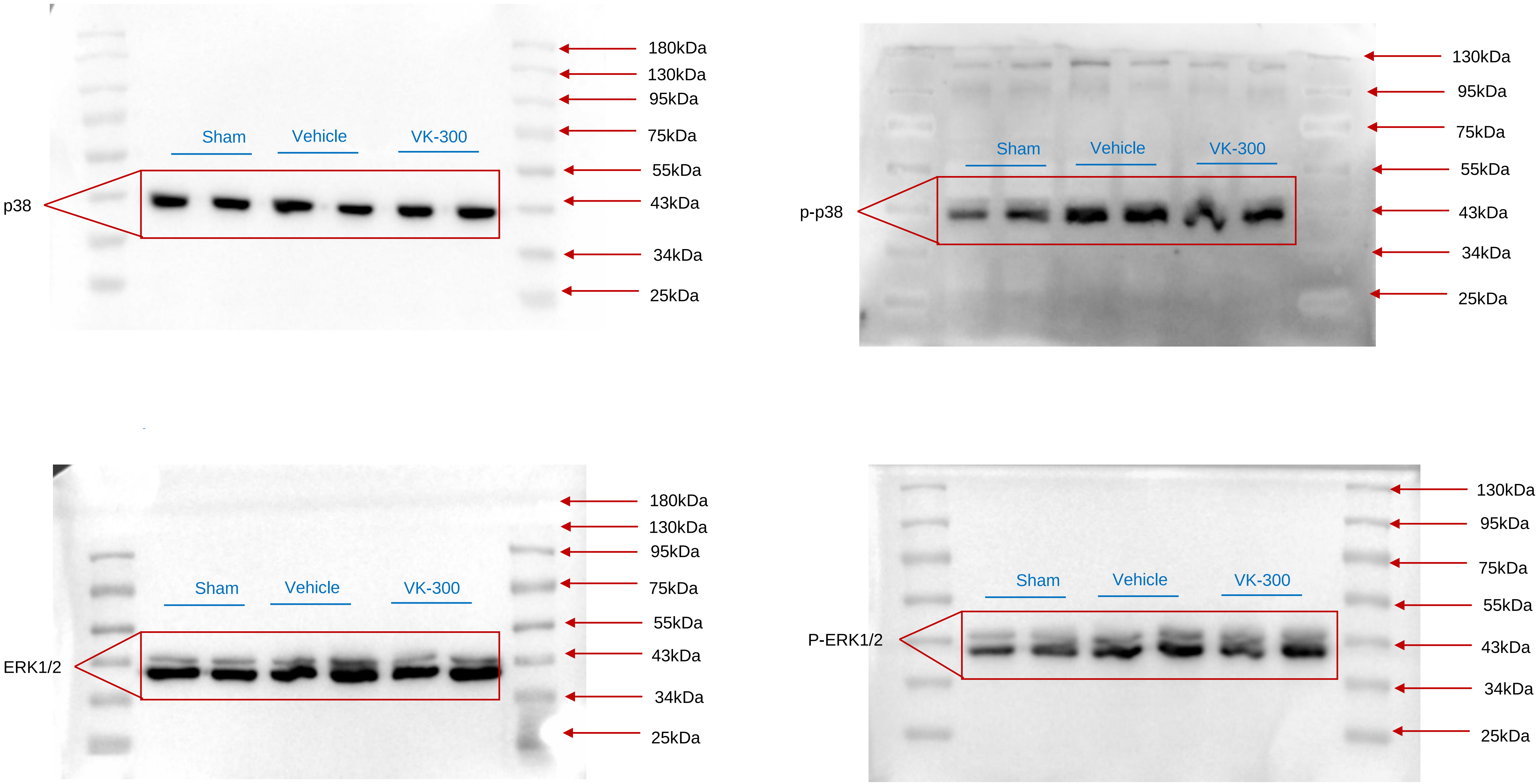

Supplementary Fig. 19: Uncropped blots referring to Supplementary Figure. 6 .

Supplementary Tables

Supplementary Table.1 357 mice for the study

Experiment	Groups	Animal numbers	Alive (Sacrifice before)	Note
Behavior test and infarct size	sham	6	6	
	vehicle	10	6	
	150	8	5	
	300	8	6	
Magnetic resonance imaging and oxidative stress in brian	sham	6	6	
	vehicle	10	6	
	150	8	6	
	300	8	6	
The structure and function of blood-brain barrier	sham	8	8	1)
	vehicle	12	8	
	150	10	9	
	300	10	9	
Pro-inflammatory cytokines in serum or ischemic penumbra of brian, immunostaining of microglia and neuron	sham	10	10	2)
	vehicle	14	10	
	150	8	6	
	300	10	8	
Patch clamp	sham	4	4	
	vehicle	6	4	
	150	6	4	
	300	6	4	
Phospho-antibody array	sham	5	5	
	vehicle	10	7	
	150	8	6	
	300	8	5	
B2R antagonist HOE140 counteracts	sham	5	5	
	vehicle	8	6	
	300	8	5	
	B1R	8	5	
	R2R	8	5	
Quantitative immunoblotting	sham	6	6	

	vehicle	16	6	
	300	12	6	
Primary microglia and flow cytometry	sham	6	6	
	vehicle	10	6	
	300	10	6	
Fluorescence imaging of VK	N/A	12	12	
Toxicity test	N/A	33	33	
Fetal C57BL/6 mice of embryonic day 16–17.5	N/A	3	3	Primary neuron
C57BL/6 mice at postnatal day 1–2	N/A	13	13	Primary mcroglia
All		357		

1) Five mice in each group were used to detect the content of EB in brain , and the other mice were used to detect the ultrastructure of ischemic penumbra by TEM.
2) Six mice in each group were used to evaluate pro-inflammatory cytokines in serum or ischemic penumbra of brian;the brian of other mice were performed frozen section.

Supplementary Table.2 The mortality rate of the animals

Groups		Animal numbers of MCAO	Alive	Mortality	Death rate (29.06)	P value is Fisher's exact probability (χ^2 test) Vs Vehicle group
Vehicle		86	61	25	29.06	-
VK	150	48	36	12	25	>0.05
(μg/kg)	300	64	52	12	18.75	>0.05

Using the contingency table chi-square test, the overall $\chi^2 = 2.101^a$. The total P value = 0.350.

A． Vehicle group have expected count less than 5. The minimum expected count is 11.88.

Supplementary Table.3 Behavioral responses and general appearance of mice treated with single dose of VK in acute toxicity study

Observation	Control (n=4)	VK (mg/kg. Body weight),				
		1.5 (n=5)	6 (n=6)	24 (n=6)	96 (n=6)	384 (n=6)
Temperature	Normal	Normal	Normal	Normal	Normal	Normal
Change In skin	No effect	No effect	No effect	No effect	No effect	No effect
Eye color change	No effect	No effect	No effect	No effect	No effect	No effect
Body weight	Normal	Normal	Normal	Decline	Decline	Decline
Diarrhea	Normal	Normal	Normal	Normal	Normal	Normal
Coma	Normal	Normal	Normal	Normal	Normal	Normal
Sedation	Normal	Normal	Normal	Normal	Normal	Normal
Drowsiness	Normal	Normal	Normal	Normal	Normal	Normal
Breathing difficulty	Normal	Normal	Normal	Normal	Normal	Normal
Tremor	Normal	Normal	Normal	Normal	Normal	Normal
Organ index	Normal	Normal	Normal	Normal	Normal	Increased
Death	Alive	Alive	Alive	Alive	Alive	Alive

Note: Continuous observation of 14 days.

Supplementary Table 4 Real-time PCR Primer sequences about inflammation

Primer	F（5' - 3'）	R（5' - 3'）
il-1β	TGTGTTTTCCTCCTTGCCTCTGAT	TGCTGCCTAATGTCCCCTTGAAT
tnf-*α*	CCAGTGTGGGAAGCTGTCTT	AAGCAAAAGAGGAGGCAACA
il-6	AAGGAGTGGCTAAGGACCAAGAC	AGTGAGGAATGTCCACAAACTGATA
il-18	GACTCTTGCGTCAACTTCAAGG	CAGGCTGTCTTTTGTCAACGA
s100a8	TTCGTGACAATGCCGTCTGA	AGGGCATGGTGATTTCCTTGT
s100a9	TGGGCTTACACTGCTCTTAC	GGTTATGCTGCGCTCCATCT
mcp1	CCACTCACCTGCTGCTACTCATTC	CTGCTGCTGGTGATCCTCTTGTAG
peg2	GACGGACCACCTCATTCTCC	CGGAGGTCCCACTTTTCCTTT
cox-1	AAGAAGCCACCTCCAGAATGC	GTCCAGGTTCCAATTGTCGT
cox-2	CTGGTGCCTGGTCTGATGATGT	AGTCTGCTGGTTTGGAATAGTTGCT
gapdh	AAGAAGGTGGTGAAGCAGG	GAAGGTGGAAGAGTGGGAGT

Supplementary Table 5 Differential expression proteins (DEPs) of Vehicle group vs. Sham group

proteinID	AveExp. Group4	AveExp. Group3	logFC	P.Value	adj.P.Value	foldchange	Regulation
IκBa (S32)	11.72223	11.05064	0.6715900	0.0000008	0.0000129	1.592827	up
NFκB(S536)	11.34937	10.97508	0.3742874	0.0028817	0.0163294	1.296199	up
P38(T180/Y182)	12.69901	12.31708	0.3819234	0.0022497	0.0163294	1.303078	up

Supplementary Table 6 Differential expression proteins (DEPs) of Vehicle group vs. VK(150 mg/kg) group

proteinID	AveExp. Group4	AveExp. Group2	logFC	P.Value	adj.P.Value	foldchange	Regulation
I*κ*Ba (S32)	11.72223	10.94502	0.7772073	0.0000000	0.0000005	1.713810	up
CHK1 (S296)	12.15346	11.66623	0.4872290	0.0004765	0.0040500	1.401750	up
NF*κ*B(S536)	11.34937	11.00320	0.3461700	0.0055926	0.0237684	1.271181	up
P38(T180/Y182)	12.69901	12.34990	0.3491030	0.0049513	0.0237684	1.273768	up
Casp7(D198)	12.96049	12.61704	0.3434511	0.0108106	0.0367559	1.268788	up
JNK(T183)	10.43686	10.13994	0.2969236	0.0171067	0.0484689	1.228522	up

Supplementary Table 7 Differential expression proteins (DEPs) of Vehicle group vs. VK (300mg/kg) group

proteinID	AveExp. Group4	AveExp. Group1	logFC	P.Value	adj.P.Value	foldchange	Regulation
I*κ*Ba (S32)	11.72223	11.05127	0.670962	0.0000001	0.0000018	1.592134	up
Casp7(D198)	12.96049	12.26104	0.699449	0.0000002	0.0000018	1.623885	up
CHK1 (S296)	12.15346	11.54825	0.605209	0.0000048	0.0000273	1.521199	up
Erk1/2(T202)	10.58169	10.11309	0.468599	0.0000938	0.0003985	1.383765	up
P38(T180/Y182)	12.69901	12.26832	0.430683	0.0002140	0.0007275	1.347872	up
NF*κ*B(S536)	11.34937	10.92714	0.422233	0.0002999	0.0008498	1.34	up
JNK(T183)	10.43686	10.02475	0.412119	0.0004310	0.0010466	1.330638	up
hsp27(S82)	9.966273	9.580248	0.386025	0.0009889	0.0021014	1.306788	up
ATM(S1981)	11.04007	10.64725	0.392827	0.0012029	0.0022722	1.312964	up
BAD(S112)	10.57765	10.23227	0.345379	0.0044398	0.0075477	1.270484	up
Casp3(D175)	10.99441	10.65267	0.341741	0.0060359	0.0093282	1.267285	up
eIF2a (S51)	9.775162	9.430753	0.344408	0.0093106	0.0131900	1.26963	up

Supplementary References

1. Chiba T, *et al.* Development of a femtomolar-acting humanin derivative named colivelin by attaching activity-dependent neurotrophic factor to its N terminus: characterization of colivelin-mediated neuroprotection against Alzheimer's disease-relevant insults in vitro and in vivo. *J Neurosci* **25**, 10252-10261 (2005).

2. Zhao Y, *et al.* TREM2 Is a Receptor for beta-amyloid that mediates microglial function. *Neuron* **97**, 1023-1031 e1027 (2018).

3. Hu Y, *et al.* High uric acid promotes dysfunction in pancreatic beta cells by blocking IRS2/AKT signalling. *Mol Cell Endocrinol* **520**, 111070 (2021).
