# Supplementary material for: Vespakinin-M, a natural peptide from Vespa magnifica, promotes functional recovery in stroke mice
Source: Commun Biol. 2022 Jan 20;5:74. doi: 10.1038/s42003-022-03024-5 (PMC8776894; doi:10.1038/s42003-022-03024-5)
Supplement: Supplementary file 3 — Description of Additional Supplementary Files [file 42003_2022_3024_MOESM3_ESM.pdf]

Description of Additional Supplementary Files

File name: Supplementary Movie 1
Description: FITC-labeled VK (100 nM) was added to each well after 15 min of incubation at 37°C, and live cell images were acquired and processed by confocal microscopy (Zeiss LSM 880). Live cell images were recorded for 90 min.

File name: Supplementary Movie 2
Description: As a negative group was FITC treated without labeled VK.

File name: Supplementary Data 1
Description: Source data underlying Figs. 1-9.
