# Supplementary material for: Vespakinin-M, a natural peptide from Vespa magnifica, promotes functional recovery in stroke mice
Source: Commun Biol. 2022 Jan 20;5:74. doi: 10.1038/s42003-022-03024-5 (PMC8776894; doi:10.1038/s42003-022-03024-5)
Supplement: Supplementary file 7 — Reporting Summary [file 42003_2022_3024_MOESM7_ESM.pdf]

nature portfolio

Corresponding author(s): Chenggui Zhang, Jidong Cheng and Yu Zhao

Last updated by author(s): Dec2,2021

Reporting Summary

Nature Portfolio wishes to improve the reproducibility of the work that we publish. This form provides structure for consistency and transparency in reporting. For further information on Nature Portfolio policies, see our Editorial Policies and the Editorial Policy Checklist.

Statistics

For all statistical analyses, confirm that the following items are present in the figure legend, table legend, main text, or Methods section.

n/a	Confirmed	
☐	☒	The exact sample size (n) for each experimental group/condition, given as a discrete number and unit of measurement
☐	☒	A statement on whether measurements were taken from distinct samples or whether the same sample was measured repeatedly
☐	☒	The statistical test(s) used AND whether they are one- or two-sided<br>*Only common tests should be described solely by name; describe more complex techniques in the Methods section.*
☒	☐	A description of all covariates tested
☐	☒	A description of any assumptions or corrections, such as tests of normality and adjustment for multiple comparisons
☐	☒	A full description of the statistical parameters including central tendency (e.g. means) or other basic estimates (e.g. regression coefficient) AND variation (e.g. standard deviation) or associated estimates of uncertainty (e.g. confidence intervals)
☐	☒	For null hypothesis testing, the test statistic (e.g. F, t, r) with confidence intervals, effect sizes, degrees of freedom and P value noted<br>*Give P values as exact values whenever suitable.*
☒	☐	For Bayesian analysis, information on the choice of priors and Markov chain Monte Carlo settings
☒	☐	For hierarchical and complex designs, identification of the appropriate level for tests and full reporting of outcomes
☒	☐	Estimates of effect sizes (e.g. Cohen's d, Pearson's r), indicating how they were calculated

Our web collection on statistics for biologists contains articles on many of the points above.

Software and code

Policy information about availability of computer code

Data collection	Images of immunofluorescence processed with Image J. Flow analysis was performed with FlowJo software (Tree Star Inc., San Carlos, CA, USA).Protein phosphorylation detection was performed with a Phospho Explorer Antibody Array (Catalog number AAM-APOSIG-1-8, Ray Biotech, USA)
Data analysis	Statistical analysis was conducted using Graph Pad Prism 8 software.

For manuscripts utilizing custom algorithms or software that are central to the research but not yet described in published literature, software must be made available to editors and reviewers. We strongly encourage code deposition in a community repository (e.g. GitHub). See the Nature Portfolio guidelines for submitting code & software for further information.

Data

Policy information about availability of data

All manuscripts must include a data availability statement. This statement should provide the following information, where applicable:

- Accession codes, unique identifiers, or web links for publicly available datasets
- A description of any restrictions on data availability
- For clinical datasets or third party data, please ensure that the statement adheres to our policy

All data generated or analyzed during this study are included in the published article and its supplementary material.

March 2021

Field-specific reporting

Please select the one below that is the best fit for your research. If you are not sure, read the appropriate sections before making your selection.

- [x] Life sciences
- [ ] Behavioural & social sciences
- [ ] Ecological, evolutionary & environmental sciences

For a reference copy of the document with all sections, see nature.com/documents/nr-reporting-summary-flat.pdf

Life sciences study design

All studies must disclose on these points even when the disclosure is negative.

Sample size	No sample size was performed. Sample size was chosen based on prior experience yielding reasonable standard deviation.
Data exclusions	Exclusion criteria in mice were performed: (1) mice with a CBF reduction of less than 70% were excluded from further assessments; (2) no neurological deficits at 3 h after MCAO/R; (3) postmortem examination shows subarachnoid hemorrhage.
Replication	All experiments were repeated multiple times (at least 3) and the results were found to be reproducible
Randomization	Animals were randomized into the treatment groups
Blinding	Blinding was used in Animal behavior tests.

Reporting for specific materials, systems and methods

We require information from authors about some types of materials, experimental systems and methods used in many studies. Here, indicate whether each material, system or method listed is relevant to your study. If you are not sure if a list item applies to your research, read the appropriate section before selecting a response.

Materials & experimental systems

n/a	Involved in the study	
[ ]	[x]	Antibodies
[ ]	[x]	Eukaryotic cell lines
[x]	[ ]	Palaeontology and archaeology
[ ]	[x]	Animals and other organisms
[x]	[ ]	Human research participants
[x]	[ ]	Clinical data
[x]	[ ]	Dual use research of concern

Methods

n/a	Involved in the study	
[x]	[ ]	ChIP-seq
[ ]	[x]	Flow cytometry
[ ]	[x]	MRI-based neuroimaging

Antibodies

Antibodies used	AKT (1:1000,4691, CST, USA), phospho-AKT (1:1000, 4046S, CST), phospho-p44/42 MAPK (Erk1/2) (Thr202/Tyr204) (1:1000, 4370S, CST), p44/42 MAPK (Erk1/2) (1:1000,4695S, CST), p38 MAPK (1:1000,8690S, CST), phospho-p38 MAPK (Thr180/Tyr182) (1:1000, 4511S, CST), NF-κB p65 (1:1000,8242S, CST), phospho-NF-κB p65 (Ser536) (1:1000,3033S, CST), IκBα (L35A5) Mouse mAb (1:1000, 4814S, CST), phospho-IκBα (Ser32) (1:1000,2859S, CST), PI3 kinase p85α (1:1000,11889S, CST), phospho-PI3 kinase p85 (Tyr458)/p55 (Tyr199) (1:1000, 17366S, CST), anti-IBA1 (1:400, 019-19741, WAKO, Japan), anti-NeuN (1:400, ab177487, Abcam, USA),PE Rat anti-Mouse CD45 (553081, BD Biosciences), FITC anti-mouse F4/80 antibody (123108, Biolegend), PerCP-Cyanine5.5 CD11b antibody (45-0112-82, Invitrogen), and APC anti-mouse I-A/I-E antibody (107614, Biolegend).
Validation	Antibodies were used according to the manufacturers' specifications.

Eukaryotic cell lines

Policy information about cell lines

Cell line source(s)	HT22 and BV2 cell lines were Mus musculus (mouse) calvaria.Primary cortical neurons (1.0 × 105 cells per well in poly-L-lysine-coated 24-well plate) were obtained from fetal C57BL/6 mice of embryonic day 16–17.5.mixed glial cultures were isolated from WT mice at postnatal day 1–3.
Authentication	HT22 and BV2 cell lines were purchased from ATCC.
Mycoplasma contamination	All cell lines tested negative for mycoplasma
Commonly misidentified lines (See ICLAC register)	N/A.

Animals and other organisms

Policy information about studies involving animals; ARRIVE guidelines recommended for reporting animal research

Laboratory animals	No.
Wild animals	Adult male C57/BL6J mice weighing 22–25 g (8-10 weeks) were obtained from Xiamen University Laboratory Animal Center.
Field-collected samples	Study did not involve samples collected from the field.
Ethics oversight	This study were designed and conducted in accordance with the approval of the Institutional Animal Care and Use Committee of Xiamen University, China (Animal Ethics no.: XMULAC20200122).

Note that full information on the approval of the study protocol must also be provided in the manuscript.

Flow Cytometry

Plots

Confirm that:

- [x] The axis labels state the marker and fluorochrome used (e.g. CD4-FITC).
- [x] The axis scales are clearly visible. Include numbers along axes only for bottom left plot of group (a 'group' is an analysis of identical markers).
- [x] All plots are contour plots with outliers or pseudocolor plots.
- [x] A numerical value for number of cells or percentage (with statistics) is provided.

Methodology

Sample preparation	Single-cell suspensions from the brain were prepared after the removal of red blood cells by ammonium-chloride-potassium (ACK) lysis buffer and isolated leukocytes in brains by 30%, 37%, 70% Percoll (GE Healthcare) density gradient centrifugation, respectively. Cell numbers were counted by a Coulter counter (Thermo Fisher). Cells were washed with buffer (PBS with 0.5% bovine serum albumin and 0.02% sodium azide) three times and subsequently stained with fluorochrome-conjugated monoclonal antibodies.
Instrument	Flow Cytometry (CytoFLEX S).
Software	CytoFLEX S and Flowjo 10.
Cell population abundance	80%~98% cells in total were collected. Total microglia (3%`7%) were identified as CD45+F4/80, M1-like microglia were identified as CD45+F4/80+CD11b+MHCII+.
Gating strategy	Using the sham group lower expression of CD45+F4/80+CD11b+MHCII+ as negative control.

- [x] Tick this box to confirm that a figure exemplifying the gating strategy is provided in the Supplementary Information.

Magnetic resonance imaging

Experimental design

Design type	MCAO/R mice were treated with VK (300 µg/mL, i.p.) at 0, 4, 22.5, 46.5 h. Mice were deeply anesthetized with 1.5% isoflurane in a mixture of 30% O2 and 69% N2O. The magnetic resonance imaging (MRI) experiments were performed on a horizontal bore 9.4 Tesla scanner operating on a Bruker Avance platform (Bruker 9.4T Biospec).
Design specifications	-The time-of-flight MRA (TOF-MRA):7 minutes<br>-Diffusion-weighted images(DWI): 3 minutes.<br>-T2-weighted imaging: 20 minutes.
Behavioral performance measures	Variables:area,volume,gray value.

Acquisition

Imaging type(s)	All data are expressed as mean±s.d.
Field strength	Diffusion,perusion and structural.
Sequence & imaging parameters	9.4 Tesla scanner
Area of acquisition	Field of view=30*30 mm, matrix size=256 *256, echo time 3=33 ms, repetition time =3500 ms.

Diffusion MRI ☒ Used ☐ Not used

Parameters: b values of 0,300,600,900,1200,1600 and 2000 s/mm2 applied in the z direction.

Preprocessing

Preprocessing software	RadiAnt DICOM Viewer.
Normalization	No.
Normalization template	No.
Noise and artifact removal	No.
Volume censoring	T2-weighted imaging was used to evaluate the infarct volume.Infarct size was indicated as the percentage of ischemic damage with respect to the ipsilateral hemispheric volume, corrected for brain edema. For each brain slice, the total areas of both hemispheres and the areas of infarction were calculated. An edema index was measured by quantifying the midline deviation (MD) calculated as the ratio between the volume of the ipsilateral hemisphere and the volume of the contralateral hemisphere.

Statistical modeling & inference

Model type and settings	No.
Effect(s) tested	No.

Specify type of analysis: ☐ Whole brain ☒ ROI-based ☐ Both

Anatomical location(s): Infarct volumes were determined from ADC maps and T2 relaxation maps by manually selecting areas of reduced ADC values or hyperintense T2 signal by a researcher blinded to the animal protocols

Statistic type for inference (See Eklund et al. 2016)	No.
Correction	No.

Models & analysis

n/a	Involved in the study
☒	☐ Functional and/or effective connectivity
☒	☐ Graph analysis
☒	☐ Multivariate modeling or predictive analysis
